# Nasal Airway Obstruction Study (NAIROS): a phase III, open-label, mixed-methods, multicentre randomised controlled trial of septoplasty versus medical management of a septal deviation with nasal obstruction

**DOI:** 10.1186/s13063-020-4081-1

**Published:** 2020-02-13

**Authors:** Katherine J. Rennie, James O’Hara, Nikki Rousseau, Deborah Stocken, Denise Howel, Laura Ternent, Mike Drinnan, Alison Bray, Leila Rooshenas, David W. Hamilton, Alison Steel, Tony Fouweather, Ann-Marie Hynes, Eva-Maria Holstein, Yemi Oluboyede, Alaa Abouhajar, Janet A. Wilson, Sean Carrie, for the NAIROS Study Group, Quentin Gardiner, Paul Nix, Samuel Leong, Naveed Kara, Jillian Morrison, Sadie Khwaja, Sangeeta Maini, Jemima Dooley, Caroline Wilson, Ian Campbell, Maria Allen, Lyndsey Lindley, Joan Mackintosh

**Affiliations:** 1 Newcastle Clinical Trials Unit, Newcastle University, Newcastle upon Tyne, NE2 4AE UK; 2 Institute of Health and Society, Newcastle University, Newcastle upon Tyne, NE2 4AX UK; 3 Freeman Hospital, Newcastle upon Tyne NHS Foundation Trust, Freeman Road, Newcastle upon Tyne, NE7 7DN UK; 4 Leeds Institute of Clinical Trials Research, University of Leeds, Leeds, LS2 9JT UK; 5 Northern Medical Physics and Clinical Engineering, Royal Victoria Infirmary, Newcastle upon Tyne NHS Foundation Trust, Newcastle upon Tyne, NE1 4LP UK; 6 Institute of Cellular Medicine, Newcastle University, Newcastle upon Tyne, NE2 4HH UK; 7 NIHR Newcastle In Vitro Diagnostics Co-operative, Newcastle University, Newcastle upon Tyne, NE2 4HH UK; 8 Bristol Population Health Science Institute, University of Bristol, Bristol, BS8 2PS UK

**Keywords:** Nasal septum, Nasal obstruction, Septoplasty, Turbinates, Mometasone furoate, Clinical trial, Cost-effectiveness, Process evaluation

## Abstract

**Background:**

Septoplasty (surgery to straighten a deviation in the nasal septum) is a frequently performed operation worldwide, with approximately 250,000 performed annually in the US and 22,000 in the UK. Most septoplasties aim to improve diurnal and nocturnal nasal obstruction. The evidence base for septoplasty clinical effectiveness is hitherto very limited.

**Aims:**

To establish, and inform guidance for, the best management strategy for individuals with nasal obstruction associated with a deviated septum.

**Methods/design:**

A multicentre, mixed-methods, open label, randomised controlled trial of septoplasty versus medical management for adults with a deviated septum and a reduced nasal airway. Eligible patients will have septal deflection visible at nasendoscopy and a nasal symptom score ≥ 30 on the NOSE questionnaire. Surgical treatment comprises septoplasty with or without reduction of the inferior nasal turbinate on the anatomically wider side of the nose. Medical management comprises a nasal saline spray followed by a fluorinated steroid spray daily for six months. The recruitment target is 378 patients, recruited from up to 17 sites across Scotland, England and Wales. Randomisation will be on a 1:1 basis, stratified by gender and severity (NOSE score). Participants will be followed up for 12 months post randomisation. The primary outcome measure is the total SNOT-22 score at 6 months. Clinical and economic outcomes will be modelled against baseline severity (NOSE scale) to inform clinical decision-making. The study includes a recruitment enhancement process, and an economic evaluation.

**Discussion:**

The NAIROS trial will evaluate the clinical effectiveness and cost-effectiveness of septoplasty versus medical management for adults with a deviated septum and symptoms of nasal blockage. Identifying those individuals most likely to benefit from surgery should enable more efficient and effective clinical decision-making, and avoid unnecessary operations where there is low likelihood of patient benefit.

**Trial registration:**

EudraCT: 2017–000893-12, ISRCTN: 16168569. Registered on 24 March 2017.

## Background

Septoplasty is surgery to straighten the nasal partition between the two nostrils (the septum). Septoplasty is a commonly conducted operation worldwide, with approximately 250,000 operations performed annually in the US and approximately 22,000 in the United Kingdom (UK) [1, 2]. Most of these are carried out for nasal blockage and associated symptoms such as a snoring and sleep disturbance.

Nasal blockage is one of the commonest complaints presenting to otolaryngologists. However, the causes may be multiple, and several may be co-existent. Septal deviation or lesions in the nasal passages, such as nasal polyps or enlarged adenoids or turbinates, may cause a ‘fixed’ sensation of blockage. ‘Fluctuating’ blockage symptoms may be caused by inflammatory conditions of the nasal epithelium such as infective or allergic rhinitis. In addition, the ‘nasal cycle’, a spontaneous physiological congestion and decongestion of the nasal cavity, compounds the challenge in characterising and assessing nasal patency [3]. The impact of the ‘nasal cycle’ can be mitigated by measuring nasal airflow following therapeutic nasal decongestion [3].

Ideally, the septum runs down the centre of the nose. If it is not straight, perhaps because of injury or a developmental anomaly, it may narrow one or both sides of the nose and obstruct airflow. A perfectly straight nasal septum in adults is rare and some degree of deviation is an accepted norm. However, in instances where there are symptoms of nasal obstruction and a concomitant deviation of the septum, patients may be offered the septoplasty operation.

On the sidewalls of the nose are ‘turbinates’, tissue structures which are rich in blood vessels and glands. Often when the septum narrows one side of the nose, it creates a larger space on the other side, into which the turbinate on that side expands. Medical management using topical nasal steroid sprays decongests the nasal lining and may lead to improvement in the symptoms of nasal blockage. However, such treatments are required on a daily, ongoing basis and in practice may not be successful. In addition, side effects of nasal dryness, irritation and bleeding may impact on treatment satisfaction and compliance. When surgery to straighten the septum is carried out, some surgeons also reduce the contralateral turbinate tissue. Potential complications of septoplasty include septal perforation, septal adhesions and bleeding [4]. Post-operative pain is common although this is reduced if sutures rather than nasal packing are used [4–6]. Patients typically are advised to take several days off work or usual activities after the operation. Septoplasty has no defined selection criteria, particularly in patients whose principal symptoms are sleep related, and clinical practice varies in different centres. The mode of action of septoplasty in sleep-related breathing disorders is not fully understood [7–9].

The effectiveness of septoplasty with or without turbinate surgery remains unclear and there is a lack of high-quality evidence of its benefit in the literature [10, 11]. Not all patients improve with surgery. Estimates of persistent septal deviation following a septoplasty procedure range from less than 6% [12] to 20% [13]. Where septoplasty fails and further surgery becomes necessary, revision rates are reported to be high [14]. There is also a lack of robust evidence about the additional benefit of turbinate surgery [11]. One study showed reduced revision rates for septoplasty when the turbinate tissue is reduced [15]; other studies report no added long-term benefit from turbinate reduction [16–18].

Currently, most septal surgery is based on subjective, unstandardised clinical impressions of the contribution of the nasal septum to patients’ symptoms. There is also no good comparative evidence regarding alternatives to septal surgery; nor about who might most benefit, to inform patients’ and doctors’ shared surgical decision-making [11].

Whilst it is recognised that that the evidence base for septoplasty is ambiguous [11], it is important to take into account the variations between men and women in relation to the operation. Firstly, septoplasty is more common in men [4, 11] and, secondly, there is a known gender influence on response to nasal-patient reported outcome measures [1].

The aim of NAIROS is to establish, and inform guidance for, the best management strategy for patients with nasal obstruction associated with a deviated nasal septum, via a randomised controlled trial (RCT) of surgery versus medical management across 17 sites in both secondary and tertiary hospitals across England, Scotland and Wales.

## Methods/design

### Aims and objectives

#### Study aim

To establish, and inform guidance for, the best management strategy for participants with nasal obstruction associated with a deviated septum, via a randomised controlled trial comparing the clinical and cost-effectiveness, of nasal septoplasty plus/minus (±) contralateral turbinate reduction versus medical management.

#### Objectives

The study objectives are split into three different aspects: clinical effectiveness, economic evaluations and mixed-method process evaluation.

##### Clinical effectiveness

To measure clinical effectiveness according to:

- Subjective self-report rating of nasal airway obstruction
- Heterogeneity of estimated treatment effect specifically according to severity of obstruction and gender
- Objective measures of nasal patency
- Number of adverse events (AEs) and additional interventions required
- Technical failure in the surgical arm
- How well those agreeing to enter the trial reflect those screened for eligibility

##### Economic evaluation

- The cost-effectiveness of each intervention
- The cost-utility with outcomes reported as incremental cost per Quality Adjusted Life Year (QALY) gained
- A longer-term economic model to assess costs and health consequences beyond 12-month follow-up period
- All economic analyses will be conducted from the perspective of the National Health Service (NHS) and participants

##### Mixed-methods process evaluation of the trial and interventions

Our mixed-method process evaluation will identify, describe, understand and address:

- Barriers to optimal recruitment, and potential solutions to address these, through integration of the QuinteT Recruitment Intervention (QRI) [19, 20]
- Participants’ and healthcare professionals’ experiences of trial participation and the interventions under evaluation
- Factors likely to influence wider implementation of trial findings

The design, measured outcomes and analysis of the process evaluation and QRI are detailed later in this manuscript.

### Trial design and duration

A multicentre, randomised controlled, open-label trial, incorporating a qualitative process and economic evaluation. Participants will be randomised on a 1:1 basis between septoplasty, with or without turbinate reduction, versus medical management (Isotonic Saline Nasal Spray (Sterimar) and Mometasone Nasal Spray) of nasal obstruction. Participants in the medical management arm will be asked to use the nasal sprays twice daily for 6 weeks, then once daily for the remainder of the 6-month period. Recruitment will take place over 20 months, with trial completion complete at 42 months (submission of final report).

#### Trial setting

**Fig. 1:**
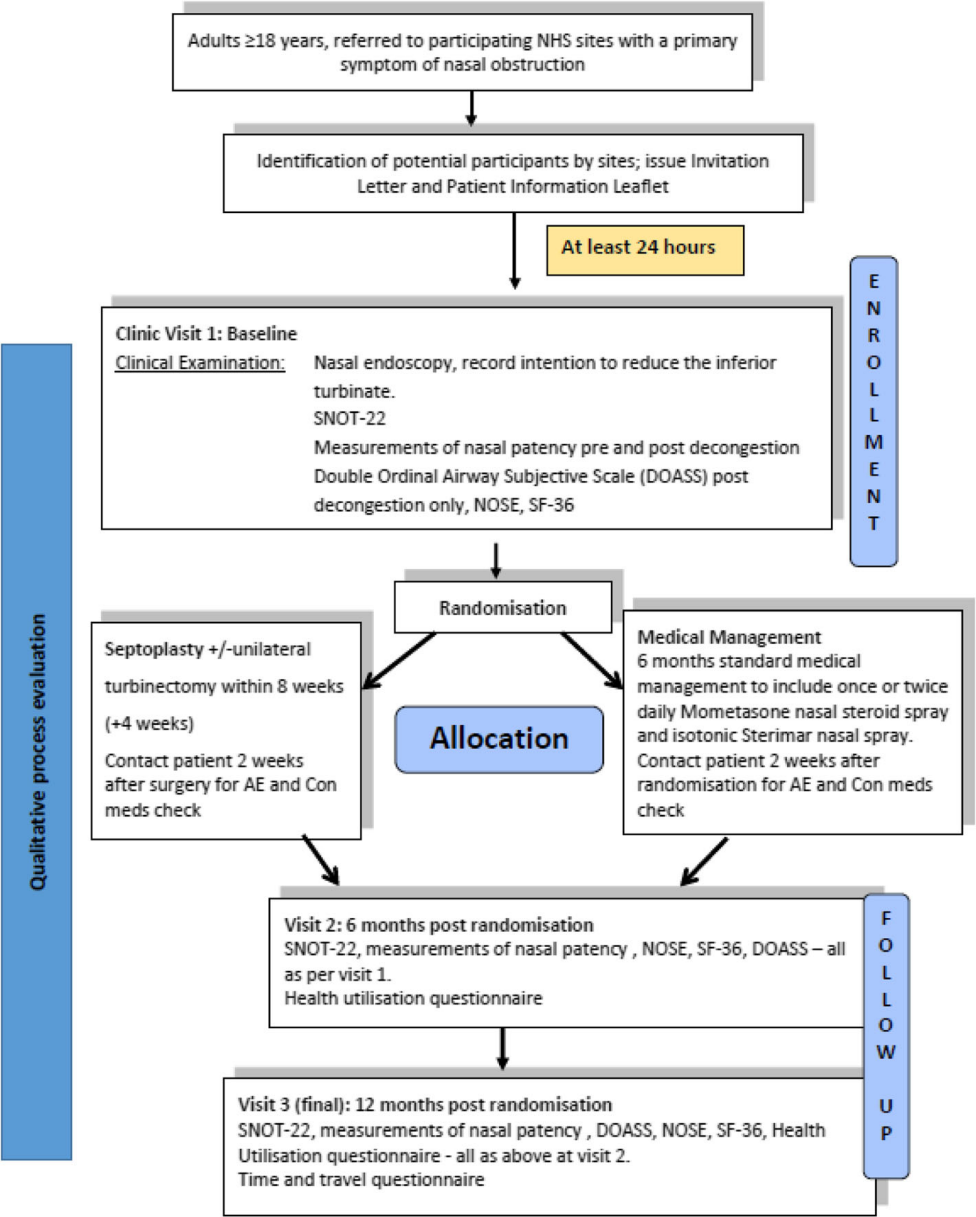
Flow Diagram of the Nasal Airway Obstruction Study (NAIROS) schedule of events

The trial will take place in 17 NHS hospitals across Scotland, England and Wales (see the ISRCTN registry number 16168569). An overview of the NAIROS schedule of events patient pathway is shown in Fig. 1.

#### Target population

Adults (aged ≥ 18 years) referred by their general practitioner (GP) to Ear, Nose and Throat (ENT) secondary care outpatient clinics who are found to have a deviated septum on nasendoscopy and reduced nasal airway as indicated by a NOSE score ≥ 30. ENT staff will also be recruited for participation in a process evaluation.

The NAIROS eligibility criteria are listed in Table 1.

**Table 1:** Nasal Airway Obstruction Study (NAIROS) eligibility criteria

Inclusion criteria	Exclusion criteria
• Adults aged ≥ 18 years • Baseline NOSE score ≥ 30 • Septal deflection at baseline visible via nasendoscopy • Capacity and willingness to provide written informed consent and complete the trial questionnaires	• Any prior septal surgery • Systemic inflammatory disease or the use of any current oral steroid treatment within the past 2 weeks • Granulomatosis with polyangiitis • Nasendoscopic evidence of unrelated associated pathology, e.g. adenoid pad, septal perforation, chronic rhinosinusitis indicated by the presence of polyposis or pus • Any history of intranasal recreational drug use within the past 6 months • Breast-feeding, pregnancy or intended pregnancy for the duration of involvement in the trial • Bleeding diathesis • Therapeutic anticoagulation (warfarin/novel oral anti-coagulant (NOAC) therapy) • Clinically significant contraindication to general anaesthesia • Patients known to be immuno-compromised • Those in whom an external bony deformity substantially contributes to the nasal obstruction

#### Participant identification, consent, screening and randomisation

Hospital researchers will proactively identify NAIROS-eligible patients through triage of referral letters of rhinology patients to the ENT department, and issue an invitation to attend a research clinic. Patients attending a research clinic will, where possible, have been sent the Patient Information Sheet (PIS) with their appointment details, and have been directed to the Patient Information Video, available at www.NAIROS.co.uk. All patients will have been given a minimum of 24 h after receiving the PIS to decide whether or not they would like to take part. The main PIS can be found in Additional file 1.

##### Consent

A delegated member of the research team will undertake informed consent discussions with the opportunity for the patient to ask any questions and discuss the trial in more detail. Patients will be invited to give informed, written consent in three stages. Firstly, consent to undergo screening (eligibility). Secondly, consent to have the discussion about the NAIROS trial with the investigator audio-recorded and their details passed onto a member of the qualitative team for a telephone interview. Finally, eligible patients are invited to give consent for the main trial, and to also give consent to potential future sharing of their anonymised data with other researchers not related to the NAIROS study. The patient Informed Consent Form can be found in Additional file 2.

##### Screening

Screening data used to assess eligibility will include:

- Clinical examination (including nasal endoscopy)
- Nasal Obstruction Symptom Evaluation Scale (NOSE) score – confirmation of total ≥ 30
- Age
- Baseline recording of four core features at endoscopy of the undecongested nose
  - ◦ The side of the maximum convexity
  - ◦ One main site of deflection on each side – anterior/ posterior/upper/lower/all)
  - ◦ Confirmation that there is no excluding inflammatory process – pus/polyps/adenoids
  - ◦ Magnitude of observer-rated airway block (< 50%; ≥ 50%)

If the participant is unable to complete the endoscopic examination without topical preparation, it can be performed after the airway assessment of the decongested nose.

The NOSE scale is a validated five-item, unifactorial self-report of nasal-block severity which has been applied in previous research and audit studies [21, 22]. The three recognised NOSE-derived categories of baseline severity used will be: 30–50 = Moderate, 55–75 = Severe, 80–100 = Extreme [22].

For NAIROS, it is anticipated that baseline severity will be the most important determinant of outcome. Those with a NOSE score of less than 30 will be excluded from NAIROS on the basis of having symptoms that are too mild to warrant inclusion.

##### Randomisation

At the baseline visit, consenting, eligible patients will be randomised on a 1:1 basis using random permuted blocks of variable length. Stratification will be by gender and baseline severity (NOSE score).

Randomisation will be administered centrally by the Newcastle Clinical Trials Unit (NCTU) web-based system. The treatment allocation is open label and the randomisation system will provide a unique trial identifier for each participant via email to a delegated member of site staff.

Participants will be randomised between:

1. Septoplasty with or without unilateral turbinate reduction
2. Medical management

#### Intervention – septoplasty

Participants allocated to the septoplasty group will undergo surgical correction of the nasal septal deviation ± unilateral reduction of the inferior turbinate on the concave side. A preliminary secondary care feasibility exercise revealed that there is considerable variation in surgical practice around the UK; rates of contralateral turbinate reduction varied between NAIROS centres from 30 to 65% of septoplasties. As a pragmatic study, NAIROS does not ask surgeons to change their usual practice in relation to contralateral turbinate reduction. NAIROS surgeons may or may not carry out unilateral turbinate surgery on the wider side, according to their assessment of the individual patient airway. Intention to reduce one turbinate will be recorded prior to randomisation. Details of the actual surgery performed will also be collected.

Participants will have a closed septoplasty, will be sutured, not packed, and will be a day case (where possible). The recommended post-operative twice-daily regimen will be of saline douche plus Naseptin Nasal Cream (or if the patient is allergic to the peanut content of Naseptin, Bactroban 2% ointment). Participants will be recommended to take a few days off work.

Nasal-steroid and saline sprays should not be part of routine standard post-operative care for NAIROS. Any additional medication required by participants will be recorded as concomitant medication.

Surgery must be carried out anytime up to 8 weeks (+ 4 weeks) after randomisation. The additional 4-week window is to allow for extenuating circumstances only, such as unexpected patient or clinical reasons that necessitate a delay in surgery. Reasons for delays to surgery will be collected and reported. The surgical intervention will be performed by surgeons who have completed their training.

#### Intervention – medical management

Patients randomised to the medical management arm will be asked to use a combination of an isotonic spray with a full twice-daily dose of a fluorinated steroid spray (mometasone furoate) which is a typical maximal medical therapy regime over a 6-month period. Preparatory work by the chief investigator indicated that most patients referred from their GP have never used this sustained combination therapy.

Sterimar Isotonic Nasal Spray dose: one spray (metred dose) into each nostril prior to using the Mometasone Nasal Spray.

Mometasone Nasal Spray dose: 100 mcg (two sprays) into each nostril twice daily for 6 weeks, followed by 100 mcg (two sprays) into each nostril once daily or 50 mcg (one spray) into each nostril twice daily for the remainder of the 6-month period.

Participants who wish to discontinue their allocated treatment, but remain in the trial, may access other treatments via the standard local NHS route. Such participants will be followed up as per their allocated treatment intervention arm. Participants in the surgical arm who wish to pursue medical treatment will not receive the trial Investigational Medicinal Product (IMP) prescription. Participants in the medical arm who wish to receive surgery and remain eligible for septoplasty should be added to the elective NHS waiting list.

#### Primary outcome measure

The primary analysis is comparison of the comprehensive, validated Sino Nasal Outcome Test–22 (SNOT-22) [23] patient-reported scores at 6 months from randomisation (− 2 weeks to + 4 weeks), with complete follow-up of participants to 12 months post randomisation. SNOT-22 is a commonly employed patient reported outcome measure in the assessment of patients with pathologies of the nose and sinuses [23–30] and was first applied to septoplasty in 2003 [31]. Our PPI work found that patient symptoms mapped better to the SNOT-22 than to the NOSE and that patients preferred the SNOT-22 measure. To maximise collection of primary outcome measure, participants who cannot attend the 6-month follow-up visit may complete SNOT-22 by post.

#### Secondary outcome measures

Secondary outcome measures can be categorised into patient-reported, safety, economic, exploratory and qualitative.

##### Patient Reported Outcome Measures (PROMs)

PROMs will be used to measure long-term change in nasal patency and quality of life:

- SNOT-22 subscales (Rhinologic, Sleep, Ear/facial pain, Psychological) at 12 months
- NOSE scale at 12 months
- Double Ordinal Airway Subjective Scale (DOASS) – administered post nasal decongestant use only at 12 months. DOASS is a subjective comparator of right and left nasal patency [32] allowing direct comparison with the spirometry measures

##### Safety outcomes

Safety outcomes will be measured by the number and characteristics of any AEs, and surgical complication/failure and re-intervention within 12 months.

##### Economic outcomes

Economic outcome measures include:

- QALY gained using the 36-item Short Form Health Survey (SF-36) questionnaire (1-week recall), further converted into QALYs using the Health Economy Survey derived from SF-36 (SF-6D) algorithm [33], at 12 months, and AEs avoided
- Use of and timing of additional interventions in primary and secondary care recorded by Health Care Utilisation Questionnaire at 6 months and 12 months
- Number of days unable to undertake usual activities recorded by Health Care Utilisation Questionnaire at 6 months and at 12 months
- Incremental cost per change in SNOT-22 at 12 months
- Costs to NHS and participants at 12 months
- Longer-term economic model to assess costs and health consequences beyond the trial

##### Exploratory outcome measures

Two of the most common objective measures of nasal patency, used in some overseas healthcare systems to assess likely benefit from septoplasty, are peak nasal inspiratory flow rate (PNIF) and nasal partitioning ratio (NPR) [34]. PNIF and NPR will be used in this trial as exploratory outcome measures.

All sites will be provided with two devices to measure two different measurements of nasal patency:

- PNIF, measured with a PNIF meter (Peak Nasal Inspiratory Flow (PNIF) Meter; GM Instruments, Kilwinning, UK)
- NPR, measured using the NV1 rhinospirometer (NV1 rhinospirometer; GM Instruments, Kilwinning, UK)

The two standard measurements will each be made before and after decongesting the nasal turbinate tissue with xylometazoline at baseline and at 6 and 12 months following randomisation.

PNIF measures the peak flow rate of air through both nostrils during inhalation using a PNIF meter with a face mask. The participant holds the mask over the nose and mouth, closes the mouth and inhales maximally (sniffs). PNIF has been shown to respond to septoplasty/turbinectomy [35] and can, therefore, be used for an overall assessment of nasal airflow impairment, and as an objective outcome measure from surgery. However, PNIF does not differentiate between the two nostrils.

Bench testing shows the NV1 rhinospirometer to be an accurate and precise objective marker of airflow symmetry [36]. The NV1 rhinospirometer has two separate channels to measure the volume of air passing through each nostril, hence deriving the NPR, the difference between right and left volumes divided by the sum. NPR ranges from symmetrical (0) to completely unilateral (±1). The NPR appears to predict the septal surgery outcome [34, 37]. Comparison of NPR during both maximal inhalation and normal tidal breathing will allow the comparative utility of these two measures to be compared and demonstrate any change in nasal function following treatment.

##### Qualitative outcomes

Qualitative outcomes will be identified through observations of training and NAIROS meetings, interviews with health professionals and participants, and audio-recording of recruitment discussions.

#### Data collection

**Fig. 2:**
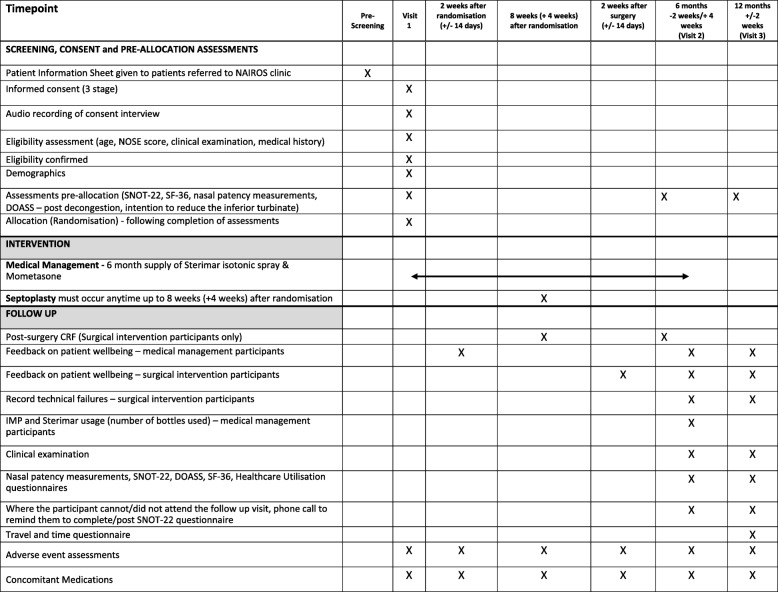
Nasal Airway Obstruction Study (NAIROS) schedule of events

The trial schedule of events is presented as a flow diagram (Fig. 1) and using the Standard Protocol Items: Recommendations for Interventional Trials (SPIRIT) Figure [38] (Fig. 2). Participants recruited to the main trial will be followed up for 12 months from the point of randomisation.

Data including the number of participants screened, approached and interested in taking part will be collected via a log completed by site staff conducting screening.

##### Assessments pre-randomisation

Eligible patients who consent to participate in the main trial will have the following outcome measures administered prior to randomisation:

- SF-36 questionnaire (1-week recall version)
- SNOT-22 questionnaire
- Nasal patency measurements (pre and post nasal decongestant):
  - ◦ PNIF
  - ◦ NPR
- Double Ordinal Airway Subjective Scale (DOASS)

##### Surgical arm data

The operating surgeon will record:

- Date of surgery
- Time into and out of theatre and duration of anaesthetic
- Highest grade of anaesthetist and surgeon
- Whether septoplasty ± unilateral turbinate reduction was carried out
- Technical aspects of the surgical procedure (flap type, closure, use of mattress sutures to septum, use of packs/splints)
- Whether there were any complications
- Discharge medication (concomitant medication)

Site nursing staff will record details of any concomitant medication and AEs during a phone call at 2 weeks after surgery has taken place, and at all scheduled trial visits.

##### Medical management arm data

As a pragmatic trial using standard treatment as part of the medical management arm, precise assessment of any mometasone furoate spray and Sterimar spray residuum will not take place. Participant compliance with the IMP does not form part of the trial monitoring plan. Participants will be asked at the 6-month follow-up visit (visit 2) to estimate how many bottles of the Sterimar and mometasone furoate spray they used.

Site nursing staff will record details of concomitant medication and any AEs during a phone call at 2 weeks post randomisation and at all scheduled trial visits.

#### Data handling and record keeping

Data will be handled, computerised, stored and archived in accordance with the General Data Protection Regulation (2018), and the latest Directive on Good Clinical Practice (GCP) (2005/28/EC). Patient-identifiable data will remain at each site and not be collected as part of the trial dataset. Patient identification on data collection tools used during screening will be through a unique sequential screening number allocated by site staff. Patients recruited to the main trial will additionally be identified by a unique trial identifier number generated by the randomisation system. Data will be transcribed and NPR files uploaded by site staff to the trial’s secure, password-limited, validated MACRO™ database (Elsevier).

The participant trial record, including completed paper data collection tools, will be archived at site for 5 years following the end of the trial. Audio-recordings will be archived for 10 years.

#### Trial compliance and withdrawal

The trial will be conducted in accordance with the Medicines for Human Use (Clinical Trials) Regulations 2004 and subsequent amendments. All parties must abide by these regulations and the International Conference on Harmonisation-Good Clinical Practice (ICH-GCP) guidelines.

Participants who withdraw their consent from the trial, or are withdrawn by the investigator, will not be replaced. All data collected up until the point of withdrawal will be retained for NAIROS research purposes, and consent will be sought for this (Additional file 2).

#### Sample size calculations

The SNOT-22 Minimal Clinically Important Difference (MCID) in the National Comparative Audit of Surgery for Nasal Polyposis and Chronic Rhinosinusitis was 8.9 [23]. Septal surgery is reported variously as showing reductions in total SNOT-22 scores above (10 points) [29] or below (4 points) this boundary [25]. In the absence of a specific figure for septoplasty MCID, NAIROS has assumed a clinically relevant reduction being at least 9 points.

Reported standard deviations (SD) of the SNOT-22 score were 18 [27] (in external septoplasty) to 24 [28] in septorhinoplasty, NAIROS assumed the larger, more conservative SD.

Sample size calculations were based on a *t* test for superiority assuming equal variance across groups, a conservative estimate given the primary analysis is based on adjustment for stratification covariates, increasing power. The target recruitment of 378 participants allows for 20% drop-out – based upon experience from our unit’s two prior septal surgery audits [10, 39]. The remaining 302 participants (151 per arm at completion), are required to show a 9-point [23] difference in overall SNOT-22 score between arms, with 90% power and 5% Type I error, assuming a SD of 24.

#### Statistical analyses

##### Primary outcome

The primary analysis is comparison of SNOT-22 scores at 6 months by randomised treatment arm (immediate surgery vs medical management). Mean overall scores will be presented by treatment group. The associated significance of any observed difference will be calculated in multivariable regression models adjusting any treatment effect by stratification factors, gender and NOSE severity at baseline. Secondary analysis of the primary outcome measure will adjust for the influence of baseline severity SNOT-22 score as a continuous covariate, planned turbinate reduction as a binary covariate and other important demographic and clinical covariates at randomisation (including, but not exclusively, age, body mass index (BMI), smoking, endoscopic features). Non-linear relationships between continuous baseline measures and outcome will be addressed by simple, and possibly more complex, fractional polynomial transformations.

The NAIROS model will generate a linear predictor score of patient outcome weighted according to the statistical importance of each covariate. Each patient’s linear predictor score will be compared against observed score for internal validation. This model will be used to explore recommendations for treatment options.

The importance of baseline severity, as a continuous distribution of NOSE score at randomisation, may be further explored graphically by Subpopulation Treatment Effect Pattern Plots (STEPP analysis) [40] to display the predicted point estimates of any treatment effect (with 95% CI) over the range of NOSE values (range 30–100 in NAIROS participants), further informing any patient selection guidance and recommendations.

Primary statistical analyses will be carried out on an intention-to-treat basis. The number of ineligible participants and reasons for ineligibility will be reported. A sensitivity analysis may be conducted and reported if the number of ineligible participants or participants not receiving the allocated treatment is excessive. Participants may choose to discontinue the treatment to which they have been allocated, and may also ask that they receive an alternative treatment as per local standard NHS care. The implication of such treatment adjustments, which typifies surgical trials, is that the intention-to-treat analysis will produce a conservative estimate of the effect of septoplasty. Non-compliance (including receiving the alternative treatment) may be addressed using an ‘as treated’ approach or complier average causal effect (CACE) approach, since the intention-to-treat analysis under non-compliance is biased when the intervention effect is large [41]. Statistical methods for withdrawal of participants, based on statistical censoring, may be considered.

Tests of heterogeneity will assess robustness of the overall treatment effect across stratification subgroups, and by intention to perform unilateral turbinate reduction.

There are no formal interim analyses of the primary outcome measure and there are no formal statistical stopping rules. Decisions regarding continuation of the trial will be made at DMC meetings held every 6 months. Decisions will be made on the basis of information presented in a statistical report that includes analysis of formal data snapshots, including safety data.

##### Analysis of secondary outcomes

Analyses of secondary outcomes will follow a broadly similar strategy. These will include the data at 6-month follow-up from the other outcomes (SNOT-22 subscales, NOSE, DOASS, SF-36) and that for all outcomes at 12-month follow-up.

Subjective scales, tabulated by arm and overall at randomisation, 6-month and 12-month follow-ups, will be compared by both summary statistics and graphical representation. Multiple regression will be used to investigate longitudinal outcome scores between treatment groups at follow-up time points. Variation between participants will be included as a random effect with an assumed normal distribution. Analysis will include the stratification factors of baseline severity and gender. Further adjusted analyses will include terms for baseline values of the scores and key demographic and clinical covariates.

Adverse events will be tabulated according to World Health Organisation (WHO) Common Terminology Criteria for Adverse Events (CTCAE) grade version 4.03. Number of severe (CTC grade 3, 4 or 5) will be reported as a proportion of all AEs. Number of participants experiencing at least one severe CTCAE will be reported as a proportion of all participants. Surgical complication/failure and re-intervention will be tabulated and will not subject to statistical testing. Technical failures from operations where widening of nasal airway was achieved yet the symptoms persist will be reported.

##### Analysis of exploratory outcomes

Three measurements each of PNIF and NPR during maximal inhalation will be made. Either the maximum (PNIF) or average (NPR) value is used.

Summary statistics will be presented for PNIF and NPR by arm and overall, at baseline, 6-month and 12-month follow-ups.

#### Mixed-method process evaluation

##### Design

The process evaluation incorporates the QRI and mixed qualitative methods. Data collection and analysis will commence during study set-up and continue throughout the trial.

Randomising patients between surgical and medical arms can be challenging. The QRI, based at Bristol University, will assist in the identification and methods of addressing such challenges. The QRI uses novel qualitative and mixed-method approaches pioneered during the National Institute for Health Research (NIHR) Health Technology Assessment (HTA)-funded ProtecT (Prostate testing for cancer and Treatment) study [42]. These methods have since been applied to several other ‘challenging’ or controversial RCTs in different clinical contexts, all of which have led to insights about recruitment issues and the development of generic and bespoke strategies to optimise recruitment [43].

The QRI will coincide with the study set-up and the first year of recruitment, using qualitative and novel methods to investigate and address recruitment barriers (objective A, below) [43–45]. Qualitative interviews will be conducted throughout the trial to investigate patients’ and clinicians’ experiences of the study procedures, interventions and barriers to implementing findings into practice (objectives B and C, below).

##### Sampling strategy

The sampling strategy is informed by current and prior experience [42, 46, 47]. In keeping with the principles of rigorous qualitative research, sampling will be responsive to the study context. In some cases fewer interviews or observations will be conducted, and in others, additional data will be required to accommodate our emerging analysis or study events. Numbers of interviews will be guided by ‘data saturation’ – continued sampling until findings become repetitious.

##### Objective A: Optimising recruitment – QRI (study set-up and first year of recruitment)

Working in close collaboration with the Trial Management Group (TMG), the QRI team will assimilate investigational and interventional approaches to understand and address recruitment difficulties in the early stages of NAIROS. The findings and implications of the QRI will continue to be implemented by the TMG and study investigators throughout the remainder of the trial recruitment period. The QRI will proceed in two iterative phases: a detailed understanding of the recruitment process will be developed in phase I, leading to tailored interventions to improve recruitment in phase II.

Phase I: understanding the recruitment process and how it operates in individual centres. A multi-faceted, flexible approach will be adopted, comprising one or more of the following methods:

- In-depth interviews, conducted with: members of the TMG (*n* = 5–10); clinicians or researchers involved in trial recruitment (*n* = 10–12); and eligible patients who have been approached to participate in the trial (*n* = 5–10). Interviews will explore views on trial processes, perceptions of equipoise, and information about how the protocol is operationalised in clinical centres
- Audio-recording and non-participant observation of consultations during which the trial is discussed with patients, enabling identification of clear and subtle obstacles to recruitment
- Mapping of eligibility and recruitment pathways – noting the point at which patients receive information about the trial, which members of the clinical team they meet, and the timing and frequency of appointments. The QRI researcher will work closely with the clinical trials unit to compose logs of potential RCT participants as they proceed through screening and eligibility phases
- Regular observation of TMG and investigator meetings to gain an overview of trial conduct and overarching challenges (logistical issues, etc.)
- Scrutiny of study documentation (e.g. PISs) to identify aspects that are unclear or potentially open to misinterpretation

Phase 2: development and implementation of recruitment strategies. Anonymised findings from Phase I will be presented to the TMG, summarising the factors that appear to be hindering recruitment. A plan of action will be devised in collaboration with the TMG if there is consensus that aspects of practice are amenable to change. Interventions will be tailored to the nature of recruitment challenges identified. Generic forms of intervention may include ‘tips’ documents on how to explain trial design and processes. Supportive feedback will be a core component of the plan of action, with the exact nature and timing of feedback dependent on the issues that arise. Centre-specific feedback may cover institutional barriers, whilst multicentre group feedback sessions may address widespread challenges. Individual confidential feedback will be offered where there is a need to discuss specific challenges or potentially sensitive issues.

##### Objectives B and C: understanding experiences of septoplasty and non-surgical management

We will investigate patients’ (*n* = 16–20) and health professionals’ (*n* = 16–20) experiences of the interventions and trial participation through qualitative interviews conducted during patient follow-up. Where possible, patients for the follow-up interviews will include those interviewed during the recruitment phase; additional participants will be recruited based on purposive and emergent criteria (e.g. patients who have declined their allocated treatment). We will identify any aspects of the care pathway which are problematic for patients or health professionals; and potential barriers and facilitators to wider acceptance and implementation of trial findings. A focus group of GPs will explore preliminary trial findings and discuss implications for primary care management of nasal obstruction. Our analysis of the implementation of study findings will be informed by Normalisation Process Theory (NPT) [48].

##### Qualitative data management and analysis

All interviews will be audio-recorded, transcribed verbatim and edited to ensure anonymity of respondents. Contemporaneous field notes from non-participant observation in clinical settings will be edited to ensure anonymity of participants. Data will be managed using NVivo software. The analysis will be conducted according to the standard procedures of rigorous qualitative analysis which we have described previously [49], including open and focussed coding, constant comparison [50], memoing [50], deviant case analysis [51] and mapping [52]. We will undertake independent coding and cross-checking and a proportion of data will be analysed collectively in ‘data clinics’ where the research team share and exchange interpretations of key issues emerging from the data. Audio-recorded recruitment consultations will be subjected to content, thematic and novel analytical approaches, including targeted conversation analysis [52] and quanti-qual appointment timing (the ‘Q-Qat method’) [53]. There will be a focus on aspects of information provision that is unclear, disrupted, or potentially detrimental to recruitment and informed consent.

#### Post-trial care

All participants who complete the NAIROS trial, or who discontinue the treatment interventions at any point, will be offered standard, local NHS care in discussion with their local investigator.

#### Indemnity

The sponsor will provide indemnity in the event that trial participants suffer negligent harm due to the management of the trial. This indemnity will be provided under the NHS indemnity arrangements for clinical negligence claims in the NHS.

#### Access to the final trial dataset

The Trial Steering Committee (TSC), Data Monitoring Committee (DMC), trial statistician, data manager and other members of the central trial team as required will have access to the full trial dataset. Individual site trial datasets will not be available to individual site investigators prior to publication of the main trial results. All requests for data should be directed to the corresponding author for consideration. Access to the anonymised final trial dataset may be available following review; we will retain exclusive use until publication of major outputs.

#### Dissemination of trial results

The results of the trial will be presented at topic-specific national or international conferences and published in a general medical journal with the monograph published by HTA. Authorship of all publications will be on a named individual authorship basis. For each publication, all individuals who fulfil the authorship definition for the publishing journal or site will be included as individually named authors. Authorship order will be decided by the chief investigator and TMG.

A lay summary of results and the HTA report will be available on the NAIROS website. Members of the Patient and Public Involvement (PPI) panel will review results and they will be involved in writing lay summaries of results for dissemination to relevant patient groups.

#### Trial monitoring

NCTU staff will monitor trial conduct and data integrity to ensure that the trial is conducted in accordance to the latest directive on GCP (2005/28/EC). This will be detailed in a Data Management Plan and a Monitoring Plan approved by the trial sponsor.

##### Safety reporting

Delegated nursing staff will interview participants to collect and record any AEs. This will take place at every trial visit (*n* = 3), and also via safety phone calls; 2 weeks after randomisation for medical management arm participants, and 2 weeks after septoplasty for surgical arm participants.

Serious adverse events (SAEs) will be assessed for any relationship to the treatment intervention (causality), and expectedness (by reference to the Reference Safety Information (RSI)) of any serious adverse reactions (SARs). Only a qualified medical doctor, delegated to do so at site, may assess the causality and expectedness of each SAE.

##### Trial Management Group

A Trial Management Group, facilitated by NCTU, will convene approximately monthly throughout the duration of the trial. Members will consist of key NCTU staff, the chief investigator, local clinical co-applicants, trial statisticians, a sponsor representative and staff representing Health Economics, Qualitative and QuinteT recruitment intervention teams.

##### Independent Data Monitoring Committee

An independent Data Monitoring Committee (DMC) has been appointed to provide an independent review of participant safety and data endpoints. The independent members comprise two statisticians and a clinician.

The DMC will meet at least annually, and report directly to the Trial Steering Committee (TSC).

##### Trial Steering Committee

A TSC has been appointed to provide overall independent supervision of the trial. Members consist of an independent chair, two independent clinicians, an independent statistician, an independent health economist and three patient representatives. The TSC will meet least annually, after a DMC meeting.

## Key changes to protocol

All substantial changes to the protocol were approved by the local UK HRA Research Ethics Committee, and standalone minor changes (version 4.1) were approved by the Health Research Authority (HRA), prior to implementation at sites. The current, full protocol is available to view on the trial funder’s website: https://fundingawards.nihr.ac.uk/search. A summary of key changes to the protocol during the trial is listed in Table 2.

**Table 2:** Key changes to the Nasal Airway Obstruction Study (NAIROS) protocol

Protocol version and date	Summary of key changes
2.0, 31 Jul 2017	• Specification of the location of the RSI within the mometasone SmPC
3.0, 20 Nov 2017	• Specification of the decongestant spray to be used alongside the nasal patency measurements (xylometazoline) and classification of the decongestant spray as a NIMP • Clarification of the exclusion criteria regarding the use of orally administered steroids and updating the exclusion criteria to exclude patients who have an external bony deformity that is likely to make a substantial contribution to the nasal obstruction
4.0, 11 Jun 2018	• Update to the mometasone RSI • Update the exclusion criteria from any history of intranasally administered recreational drug use to any history of intranasal recreational drug use within the past 6 months • Clarify the clinical examination procedure to state that patients who request local anaesthetic for nasal endoscopy may have the nasal endoscopy assessment carried out after the other trial assessments have been completed • Clarification of the timing for the surgical intervention to state that patients randomised to septoplasty must have their septoplasty anytime within 8 weeks of randomisation
4.1, 21 Dec 2018	Addition of a 4-week window to the timeline for surgery for use in extenuating circumstances (i.e. 8 weeks + 4 weeks)
5.0, 16 Jan 2019	• Change the window for the 6-month visit from ± 2 weeks to − 2 weeks/+ 4 weeks to maximise collection of the primary outcome measure • Clarification of management of patients between the 6-month and 12-month follow-up visits • Clarification of management and options for participants who wish to discontinue with their allocated treatment and explore other surgical or medical treatments as part of standard NHS care • State that discontinuation of allocated treatment does not constitute withdrawal from the trial. Update to the RSI for the surgical intervention

## Discussion

There is a paucity of evidence underlying the indications for septoplasty in the UK. At present, the decision to perform septoplasty is based on the clinician’s subjective estimation of the impact on the affected nasal airway caused by a deviated septum. In addition, there is a lack of evidence of the impact of a standardised topical medical treatment regimen on the nasal airway in the presence of a septal deflection.

At a time of rising healthcare costs and increasing scrutiny on the requirement to justify clinical interventions there is an urgent need to answer these questions. The aim of NAIROS is to perform a RCT to compare surgical treatment to a standardised dual medical therapy (Sterimar spray and mometasone spray) and estimate the effectiveness based on subjective nasal symptoms, objective airway measurements and the impact on quality of life. Furthermore, a number of other interactions will be measured at baseline, 6 and 12 months following randomisation. The impact of known covariates including sex, turbinate enlargement and subjective degree of nasal obstruction will be assessed.

NAIROS is a pragmatic ‘real-world’ trial, researching a common surgical intervention against a contemporary comparator in such a way that the results will be generalisable to NHS patients in whom it is offered. However, limitations are anticipated in both treatment arms. In the surgical arm clinicians may vary in their assessment and documentation of the nasal septum deflection. It is also recognised that there are shortcomings in objective measurements of the nasal airway [54]. In the medical arm we are not monitoring quantities of nasal-steroid used and instead relying on patient-reported use.

NAIROS will also compare the cost-effectiveness, to the patient and the NHS, of both the medical and surgical arms of the trial. The challenges and barriers to patient recruitment will be analysed by the Quintet Recruitment Intervention with a view to identifying and minimising these. A qualitative evaluation will explore the views of participants and staff and their experience of the intervention to enable us to shape guidelines and inform clinical decision-making in patients with a deviated nasal septum. The overarching aim will be to shape future guidance on the management of a deviated nasal septum in an NHS setting.

## Trial status

The NAIROS trial is currently working to protocol version 5.0, dated 16 January 2019. Recruitment began on 18 January 2018, and is due to end on 31 January 2020.

## Supplementary information

**Additional file 1.** Patient Information Sheet, V5.1 dated 27 March 2019.

**Additional file 2.** Nasal Airway Obstruction Study (NAIROS) Main Informed Consent Form, V3.0 dated 16 Jan 2019.

**Additional file 3.** SPIRIT 2013 Checklist: Recommended items to address in a clinical trial protocol and related documents.

## Data Availability

Data-sharing is not applicable to this article as no datasets were generated or analysed for this article. The main PIS and main Informed Consent Form are presented as Additional files 1 and 2, respectively.

## Abbreviations

AE: Adverse event
BMI: Body Mass Index
CACE: Complier Average Causal Effect
CRN: Clinical Research Network
CTCAE: Common Terminology Criteria for Adverse Events
DMC: Data Monitoring Committee
DOASS: Double Ordinal Airway Subjective Scale
ENT: Ear, Nose and Throat
EudraCT: European Union Drug Regulating Authorities Clinical Trials
GCP: Good Clinical Practice
GDPR: General Data Protection Regulation
GP: General practitioner
HRA: Health Research Authority
HTA: Health Technology Assessment
ICH: International Conference on Harmonisation of technical requirements for registration of pharmaceuticals for human use
IMP: Investigational Medicinal Product
ISRCTN: International Standard Randomised Controlled Trials Number
MCID: Minimally Important Clinical Difference
MHRA: Medicines and Healthcare products Regulatory Agency
NAIROS: Nasal Airway Obstruction Study
NCTU: Newcastle Clinical Trials Unit
NHS: National Health Service
NIHR: National Institute for Health Research
NOAC: Novel oral anti-coagulant
NOSE: Nasal Obstruction and Septoplasty Effectiveness
NPR: Nasal partitioning ratio
NPT: Normalisation Process Theory
PIS: Patient Information Sheet
PNIF: Peak nasal inspiratory flow rate
PPI: Patient and Public Involvement
PROM: Patient Reported Outcome Measure
QALY: Quality Adjusted Life Year
Q-Qat: Quanti-Qual appointment timing
QRI: QuinteT Research Intervention
QuinteT: Qualitative Research Integrated within Trials
RCT: Randomised controlled trial
REC: Research Ethics Committee
RSI: Reference Safety Information
SAE: Serious adverse event
SAR: Serious adverse reaction
SD: Standard deviation
SF-36: 36-item Short Form Health Survey
SF-6D: Health Economy Survey derived from SF-36
SNOT-22: Sino-Nasal Outcome Test 22
SPIRIT: Standard Protocol Items-Recommendations for Interventional Trials
TMG: Trial Management Group
TSC: Trial Steering Committee
UK: United Kingdom
US: United States of America
WHO: World Health Organisation

## References

[1] Bhattacharyya N (2010). Ambulatory sinus and nasal surgery in the United States: demographics and perioperative outcomes. Laryngoscope.

[2] NHS Digital (Hospital Episode Statistics, Admitted Patient Care – England 2014–15). https://digital.nhs.uk/data-and-information/publications/statistical/hospital-admitted-patient-care-activity/hospital-episode-statistics-admitted-patient-care-england-2014-15. Accessed 1 Oct 2016.

[3] Moore M, Eccles R (2012). Nasal patency: problems in obtaining standard reference values for the surgeon. J Laryngol Otol.

[4] Dabrowska-Bien J, Skarzynski PH, Gwizdalska I, Łazȩcka K (2018). Complications in septoplasty based on a large group of 5639 patients. Eur Arch Otorhinolaryngol.

[5] Banglawala SM, Gill M, Sommer DD, Psaltis A, Schlosser R, Gupta M (2013). Is nasal packing necessary after septoplasty? A meta-analysis. Int Forum Allergy Rhinol.

[6] Hong CJ, Monteiro E, Badhiwala J, Lee J, de Almeida JR, Vescan A (2016). Open versus endoscopic septoplasty techniques: a systematic review and meta-analysis. Am J Rhinol Allergy.

[7] Bugten V, Nilsen AH, Thorstensen WM, Moxness MH, Amundsen MF, Nordgård S (2016). Quality of life and symptoms before and after nasal septoplasty compared with healthy individuals. BMC Ear Nose Throat Disord.

[8] Sabbe AV, De Medts J, Delsupehe K (2017). Surgical treatments for snoring. B-ENT.

[9] Awad MI, Kacker A (2018). Nasal obstruction considerations in sleep apnea. Otolaryngol Clin N Am.

[10] Enache A, Lieder A, Issing W (2014). Nasal septoplasty with submucosal diathermy to inferior turbinates improves symptoms at 3 months postoperatively in a study of one hundred and one patients. Clin Otolaryngol.

[11] van Egmond M, Rovers MM, Tillema AH, van Neerbeek N (2018). Septoplasty for nasal obstruction due to a deviated nasal septum in adults: a systematic review. Rhinology.

[12] Al-Raggad DK, El-Jundi AM, Al-Momani OS, Al-Serhan MM, Nawasrah OO, Qhawi MA (2007). Suturing of the nasal septum after septoplasty, is it an effective alternative to nasal packing?. Saudi Med J.

[13] Dursun E, Battal B (2009). Clinical outcomes of nasal septal surgery at high altitude. Eur Arch Otorhinolaryngol.

[14] Gillman GS, Egloff AM, Rivera-Serrano CM (2014). Revision septoplasty: a prospective disease-specific outcome study. Laryngoscope.

[15] Karlsson TR, Shakeel M, Supriya M, Ram B, Ah-See KW (2015). Septoplasty with concomitant inferior turbinate reduction reduces the need for revision procedure. Rhinology.

[16] Siegel NS, Gliklich RE, Taghizadeh F, Chang Y (2000). Outcomes of septoplasty. Otolaryngol Head Neck Surg.

[17] Nunez DA, Bradley PJ (2000). A randomised clinical trial of turbinectomy for compensatory turbinate hypertrophy in patients with anterior septal deviations. Clin Otolaryngol Allied Sci.

[18] Devseren NO, Ecevit MC, Erdag TK, Ceryan K (2011). A randomized clinical study: outcome of submucous resection of compensatory inferior turbinate during septoplasty. Rhinology.

[19] Donovan J, Mills N, Smith M, Brindle L, Jacoby A, Peters T (2002). Quality improvement report: improving design and conduct of randomised trials by embedding them in qualitative research: ProtecT (prostate testing for cancer and treatment) study. Commentary: presenting unbiased information to patients can be difficult. BMJ.

[20] Donovan JL, Rooshenas L, Jepson M, Elliott D, Wade J, Avery K (2016). Optimising recruitment and informed consent in randomised controlled trials: the development and implementation of the Quintet Recruitment Intervention (QRI). Trials.

[21] Stewart MG, Smith TL, Weaver EM, Witsell DL, Yueh B, Hannley MT (2004). Outcomes after nasal septoplasty: results from the Nasal Obstruction Septoplasty Effectiveness (NOSE) study. Otolaryngol Head Neck Surg.

[22] Lipan MJ, Most SP (2013). Development of a severity classification system for subjective nasal obstruction. JAMA Facial Plast Surg.

[23] Hopkins C, Gillett S, Slack R, Lund VJ, Browne JP (2009). Psychometric validity of the 22-item sinonasal outcome test. Clin Otolaryngol.

[24] Browne JP, Hopkins C, Slack R, Cano SJ (2007). The Sino-Nasal Outcome Test (SNOT): can we make it more clinically meaningful?. Otolaryngol Head Neck Surg.

[25] Hytönen ML, Lilja M, Mäkitie AA, Sintonen H, Roine RP (2012). Does septoplasty enhance the quality of life in patients?. Eur Arch Otorhinolaryngol.

[26] Abdalla S, Alreefy H, Hopkins C (2012). Prevalence of Sinonasal Outcome Test (SNOT-22) symptoms in patients undergoing surgery for chronic rhinosinusitis in the England and Wales National Prospective Audit. Clin Otolaryngol.

[27] Phillips PS, Stow N, Timperley DG, Sacks R, Srubiski A, Harvey RJ, Marcells GN (2011). Functional and cosmetic outcomes of external approach septoplasty. Am J Rhinol Allergy.

[28] Poirrier AL, Ahluwalia S, Goodson A, Ellis M, Bentley M, Andrews P (2013). Is the Sino-Nasal Outcome Test-22 a suitable evaluation for septorhinoplasty?. Laryngoscope.

[29] Pannu KK, Chadha S, Kaur IP (2009). Evaluation of benefits of nasal septal surgery on nasal symptoms and general health. Indian J Otolaryngol Head Neck Surg.

[30] Lal D, Rounds AB, Divekar R (2016). Gender-specific differences in chronic rhinosinusitis patients electing endoscopic sinus surgery. Int Forum Allergy Rhinol.

[31] Buckland JR, Thomas S, Harries PG (2003). Can the Sino-nasal Outcome Test (SNOT-22) be used as a reliable outcome measure for successful septal surgery?. Clin Otolaryngol Allied Sci.

[32] Boyce JM, Eccles R (2006). Assessment of subjective scales for selection of patients for nasal septal surgery. Clin Otolaryngol.

[33] Brazier J, Roberts J, Deverill M (2002). The estimation of a preference-based measure of health from the SF-36. J Health Econ.

[34] Cuddihy PJ, Eccles R (2003). The use of nasal spirometry as an objective measure of nasal septal deviation and the effectiveness of septal surgery. Clin Otolaryngol Allied Sci.

[35] Balikci HH, Gurdal MM (2014). Use of peak nasal inspiratory flowmetry and nasal decongestant to evaluate outcome of septoplasty with radiofrequency coblation of the inferior turbinate. Rhinology.

[36] Owens D, Moore M, Craven C, Magurean C, Backhouse S, Whittet H (2011). The accuracy and reproducibility of rhinospirometry in detecting flow asymmetry in a nasal cavity model. Eur Arch Otorhinolaryngol.

[37] Fyrmpas G, Kyrmizakis D, Vital V, Constantinidis J (2011). The value of bilateral simultaneous nasal spirometry in the assessment of patients undergoing septoplasty. Rhinology.

[38] Chan A.-W., Tetzlaff J. M., Gotzsche P. C., Altman D. G., Mann H., Berlin J. A., Dickersin K., Hrobjartsson A., Schulz K. F., Parulekar W. R., Krleza-Jeric K., Laupacis A., Moher D. (2013). SPIRIT 2013 explanation and elaboration: guidance for protocols of clinical trials. BMJ.

[39] Arunachalam PS, Kitcher E, Gray J, Wilson JA (2001). Nasal septal surgery: evaluation of symptomatic and general health outcomes. Clin Otolaryngol Allied Sci.

[40] Bonetti M, Gelber RD (2004). Patterns of treatment effects in subsets of patients in clinical trials. Biostatistics.

[41] Dumville JC, Torgerson DJ, Hewitt CE (2006). Reporting attrition in randomised controlled trials. BMJ.

[42] Rousseau N, McColl E, Newton J, Grimshaw J, Eccles M (2003). Practice based, longitudinal, qualitative interview study of computerised evidence based guidelines in primary care. BMJ.

[43] Rubie I, Haighton C, O'Hara J, Rousseau N, Steen N, Stocken DD (2015). The NAtional randomised controlled Trial of Tonsillectomy IN Adults (NATTINA): a clinical and cost-effectiveness study: study protocol for a randomised control trial. Trials.

[44] Rapley T (2008). Distributed decision making: the anatomy of decisions-in-action. Sociol Health Illn.

[45] Donovan JL, Paramasivan S, de Salis I, Toerien M (2014). Clear obstacles and hidden challenges: understanding recruiter perspectives in six pragmatic randomised controlled trials. Trials.

[46] Donovan JL, Brindle L, Mills N (2002). Capturing users’ experiences of participating in cancer trials. Eur J Cancer Care (Engl).

[47] May CR, Finch T, Ballini L, MacFarlane A, Mair F, Murray E (2011). Evaluating complex interventions and health technologies using normalization process theory: development of a simplified approach and web-enabled toolkit. BMC Health Serv Res.

[48] Paleri V, Wood J, Patterson J, Stocken DD, Cole M, Vale L (2016). A feasibility study incorporating a pilot randomised controlled trial of oral feeding plus pre-treatment gastrostomy tube versus oral feeding plus as-needed nasogastric tube feeding in patients undergoing chemoradiation for head and neck cancer (TUBE trial): study protocol. Pilot Feasibility Stud.

[49] Glaser B (1965). The constant comparative method of qualitative analysis. Soc Probl.

[50] Seale C. The quality of qualitative research. London: Sage; 1999.

[51] Charmaz K. Constructing grounded theory: a practical guide through qualitative analysis. London: Sage; 2006.

[52] Wade J, Donovan JL, Lane JA, Neal DE, Hamdy FC (2009). It’s not just what you say, it’s also how you say it: opening the ‘black box’ of informed consent appointments in randomised controlled trials. Soc Sci Med.

[53] Paramasivan S, Strong S, Wilson C, Campbell B, Blazeby JM, Donovan JL (2015). A simple technique to identify key recruitment issues in randomised controlled trials: Q-QAT—Quanti-Qualitative Appointment Timing. Trials.

[54] Calder NJ, Swan IR (2007). Outcomes of septal surgery. J Laryngol Otol.

[55] Townsend D, Mills N, Savovic J, Donovan JL (2015). A systematic review of training programmes for recruiters to randomised controlled trials. Trials.

