# Supplementary figures and images for: Nasal Airway Obstruction Study (NAIROS): a phase III, open-label, mixed-methods, multicentre randomised controlled trial of septoplasty versus medical management of a septal deviation with nasal obstruction
Source: Trials. 2020 Feb 13;21:179. doi: 10.1186/s13063-020-4081-1 (PMC7020359; doi:10.1186/s13063-020-4081-1)

**Appendix 1. Patient Information Sheet, V5.1 dated 27 MARCH 2019**

**
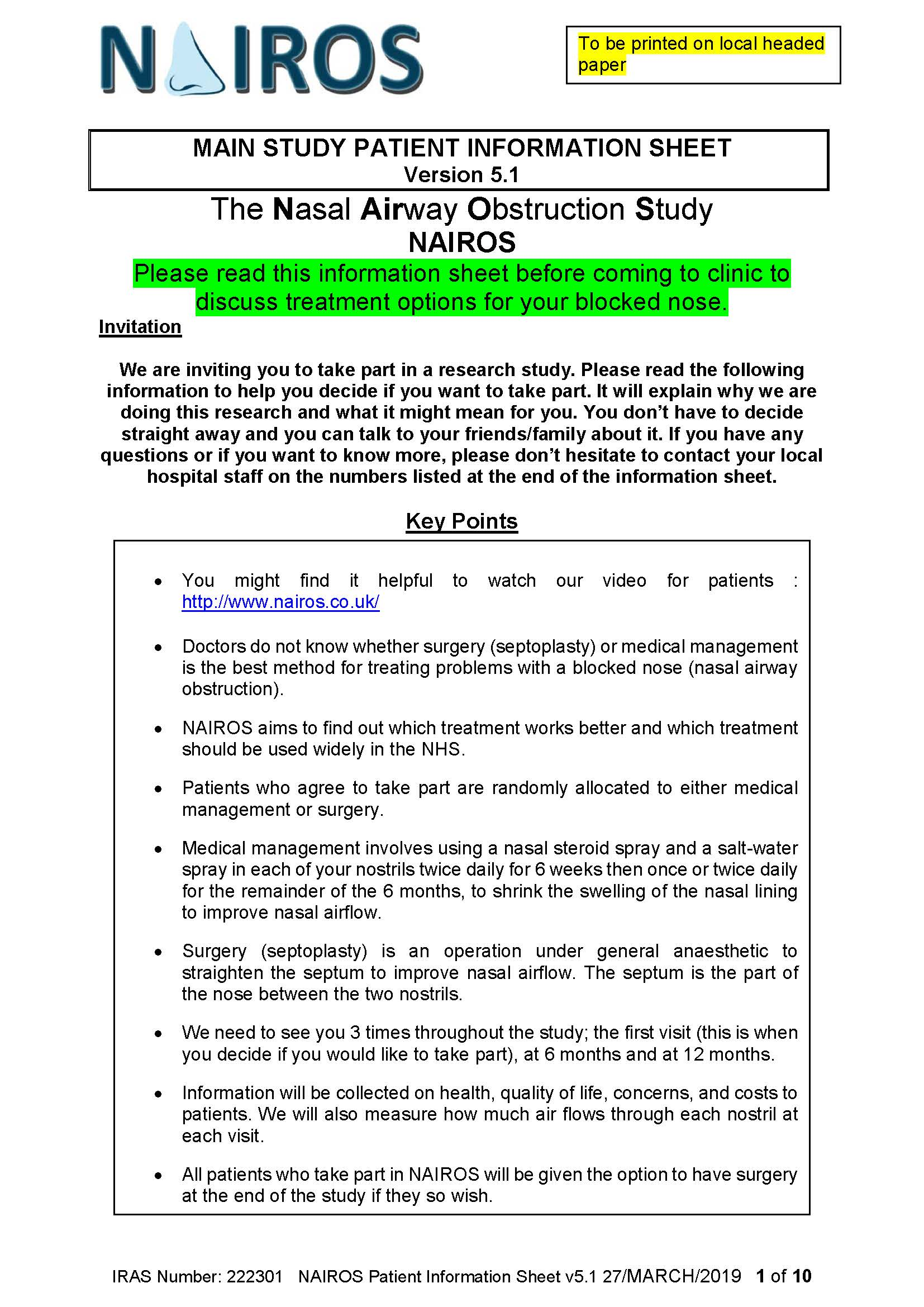
**

**
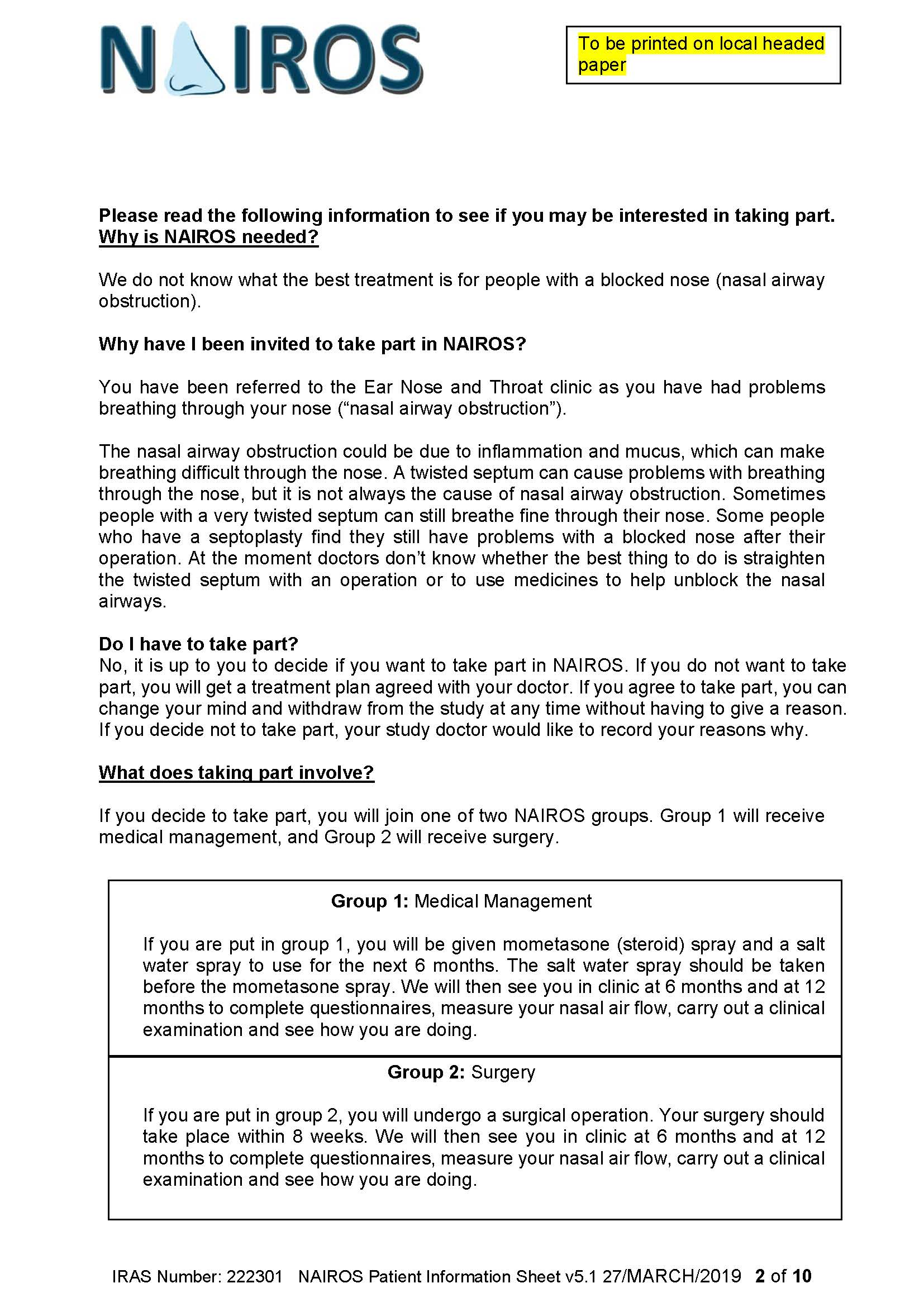
**

**
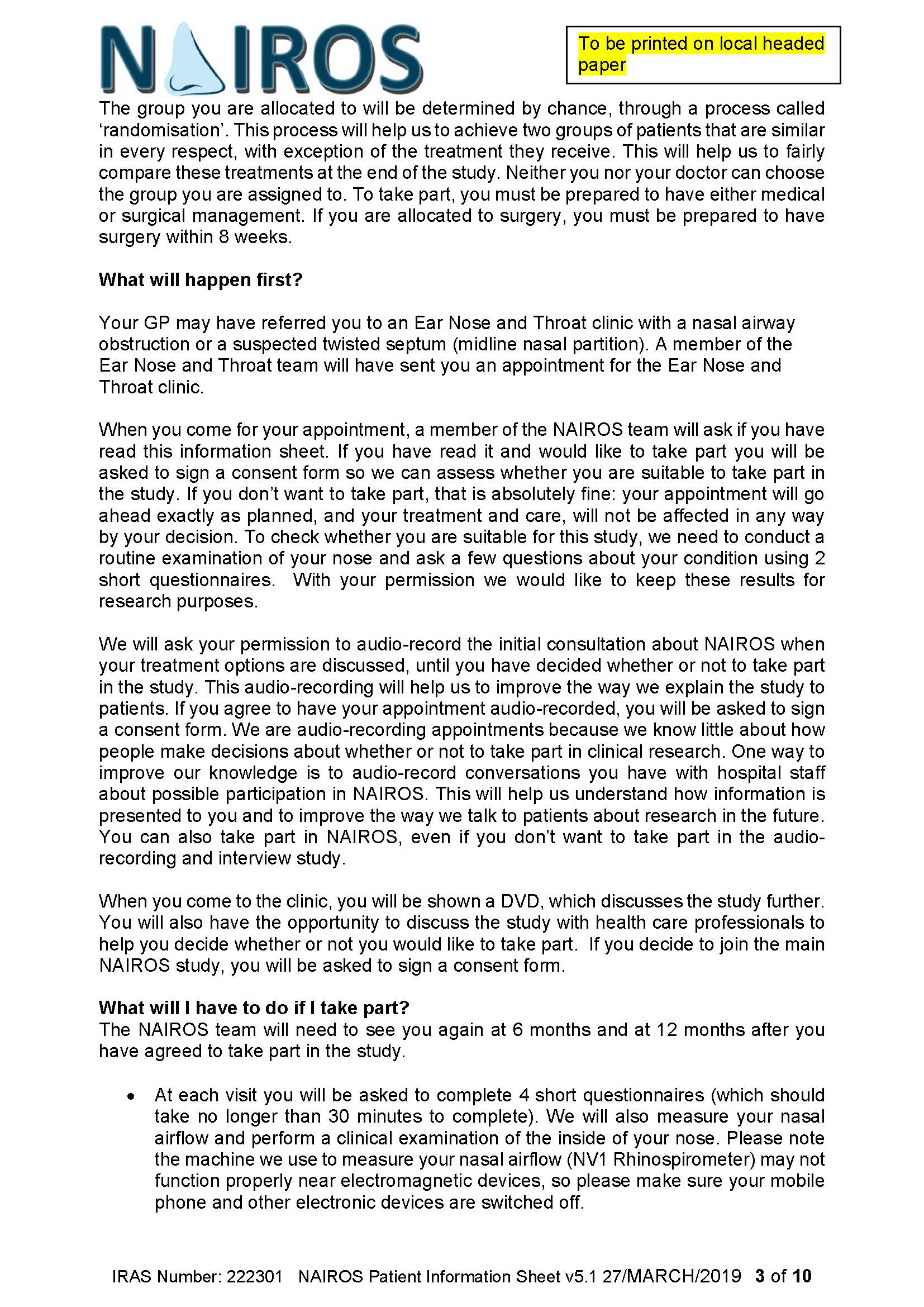
**

**
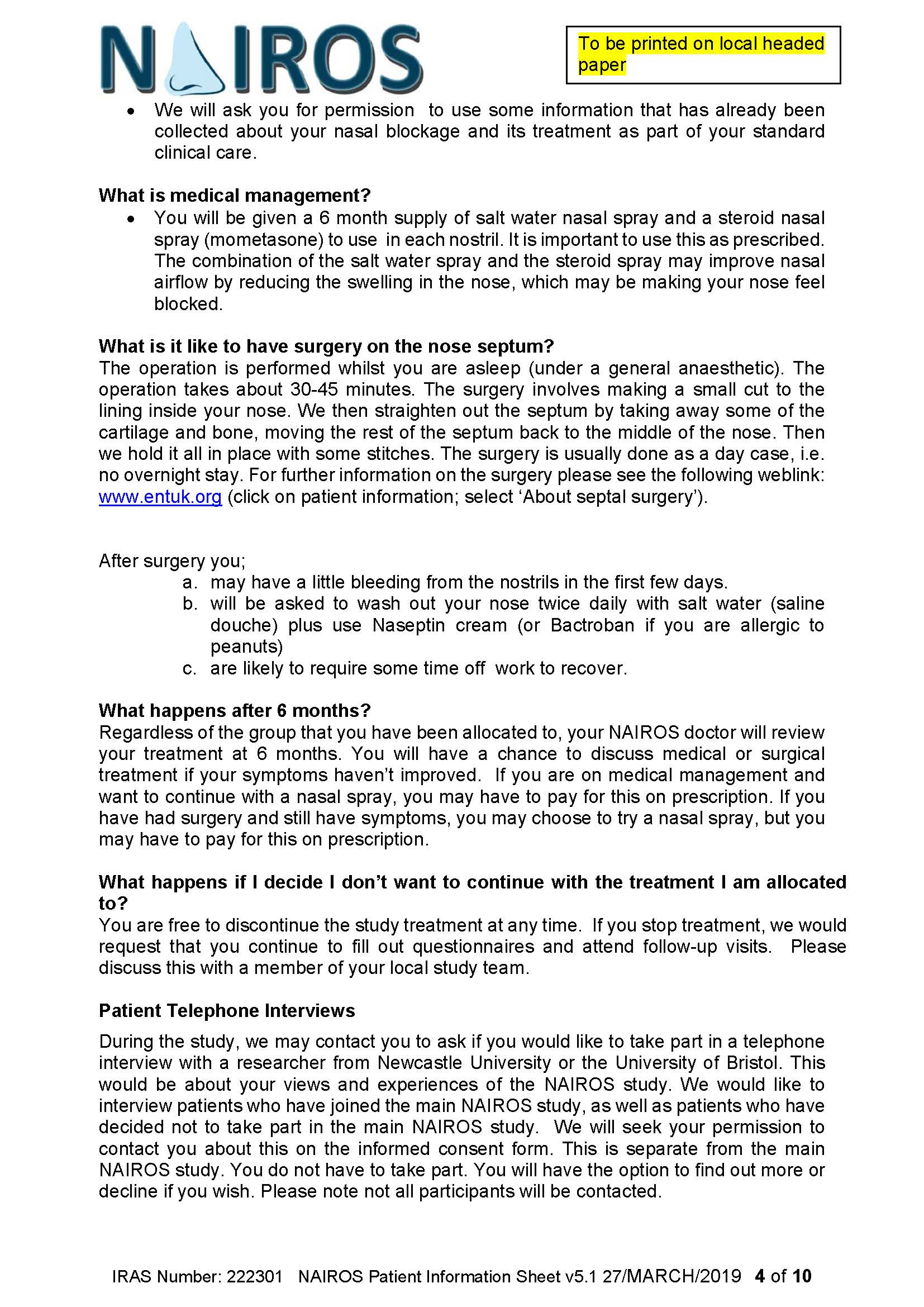
**

**
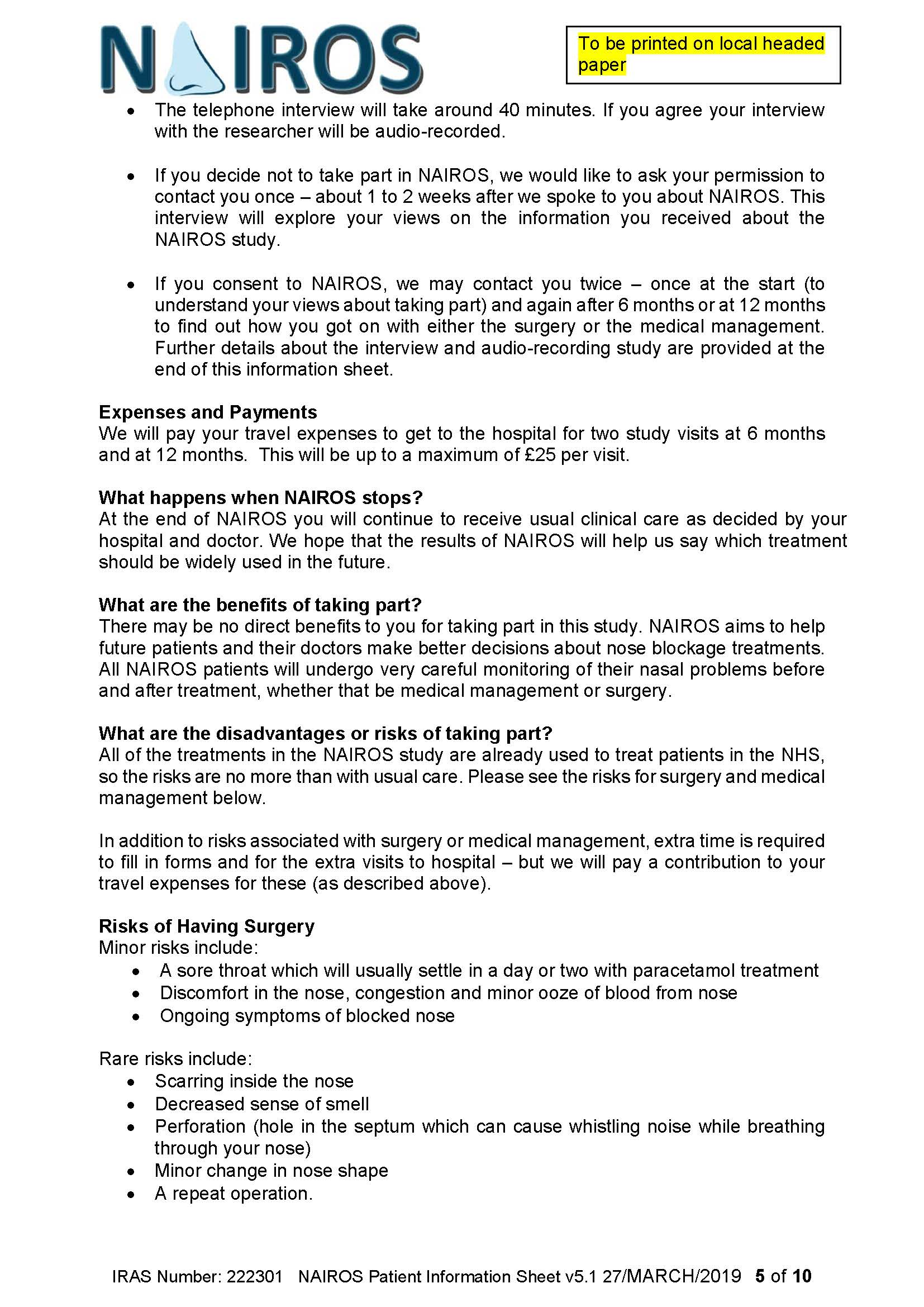
**

**
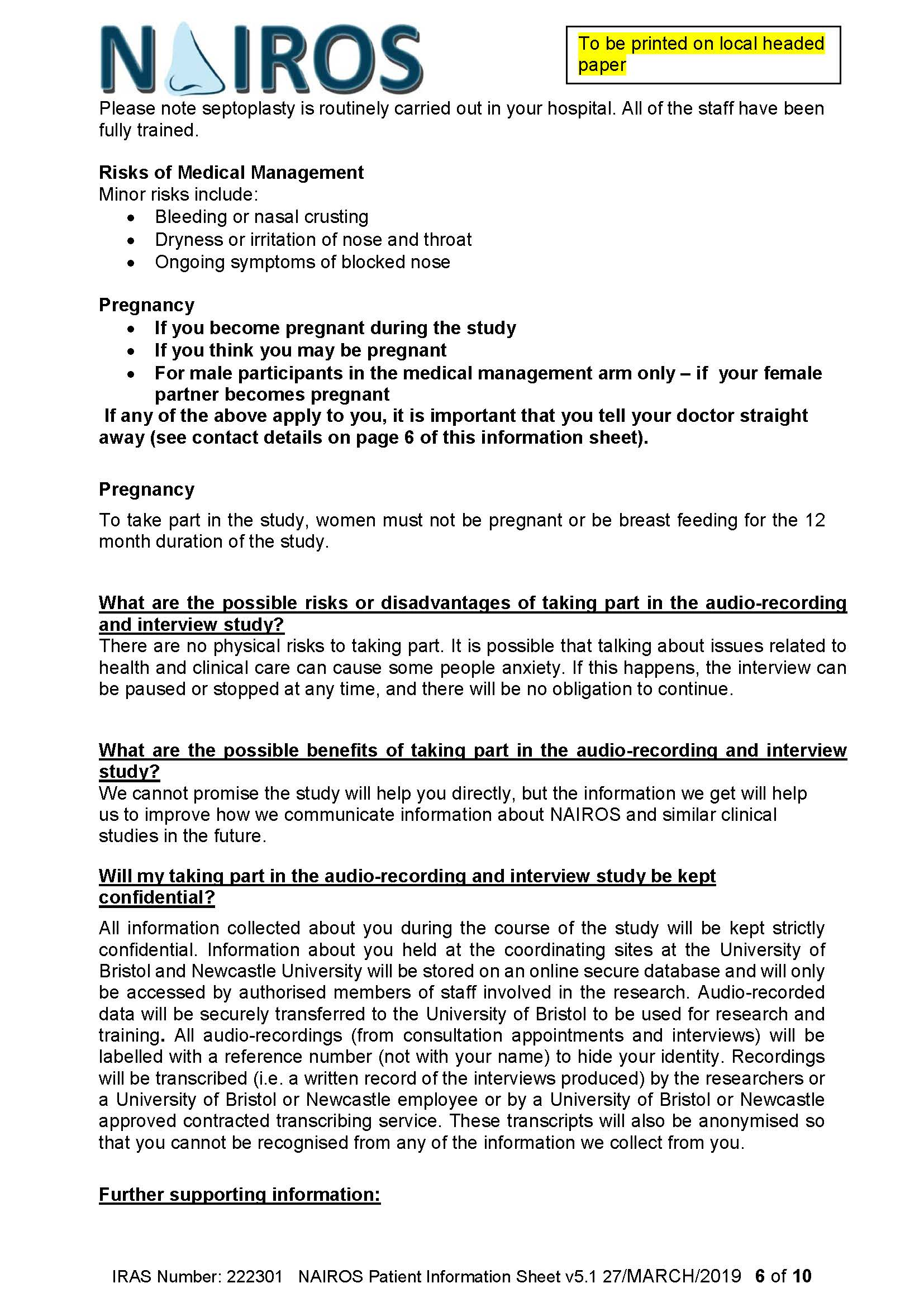
**

**
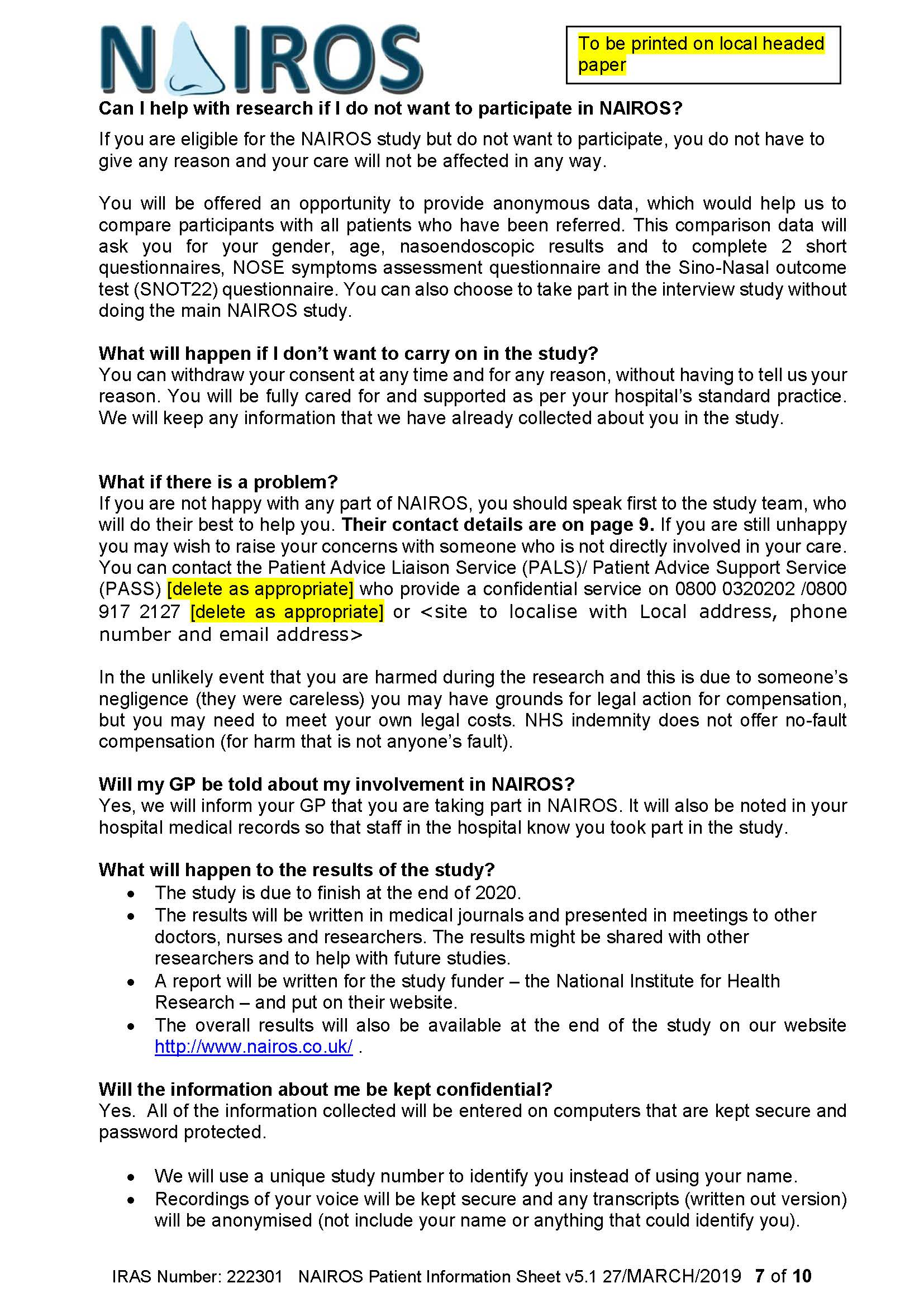
**

**
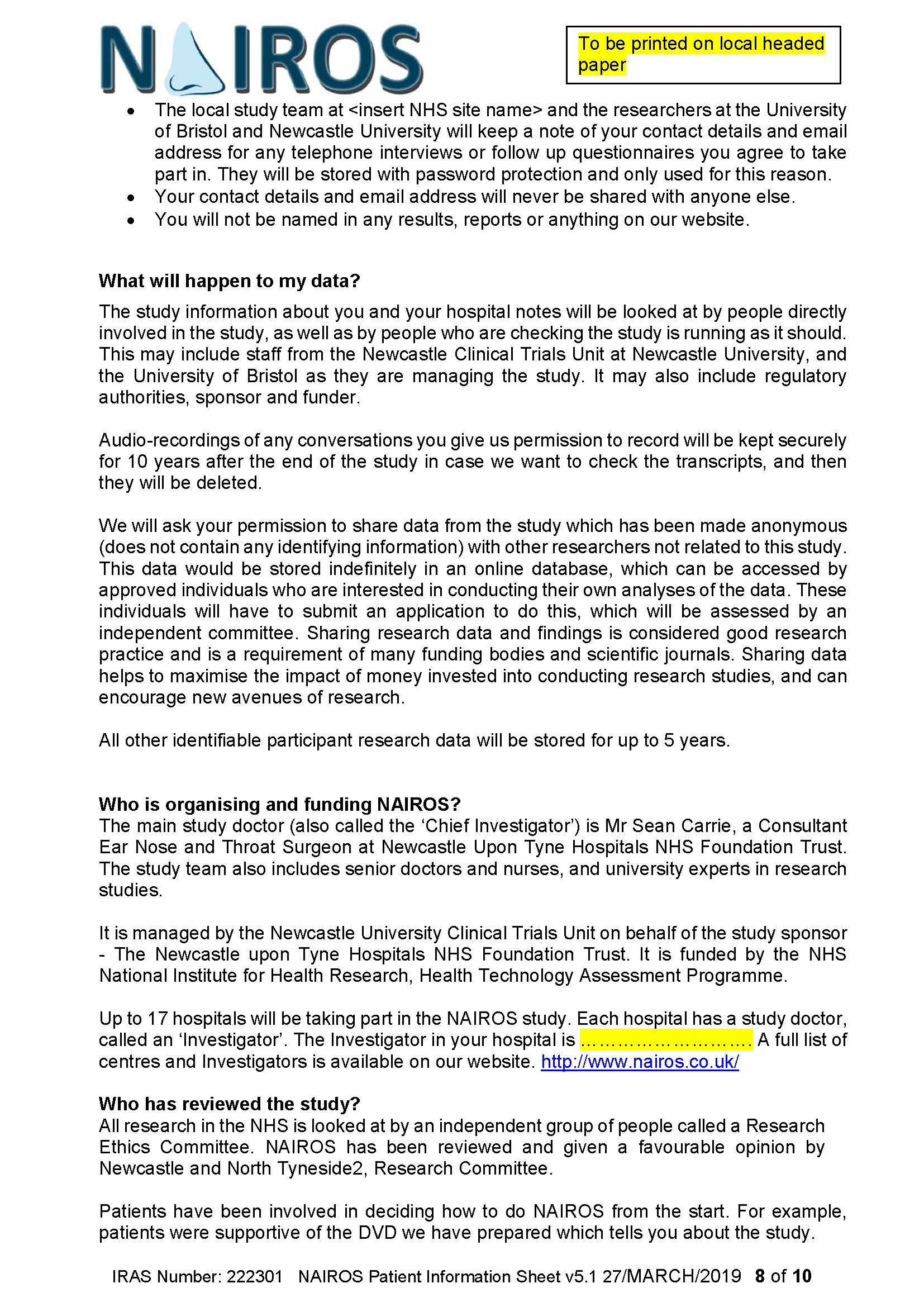
**

**
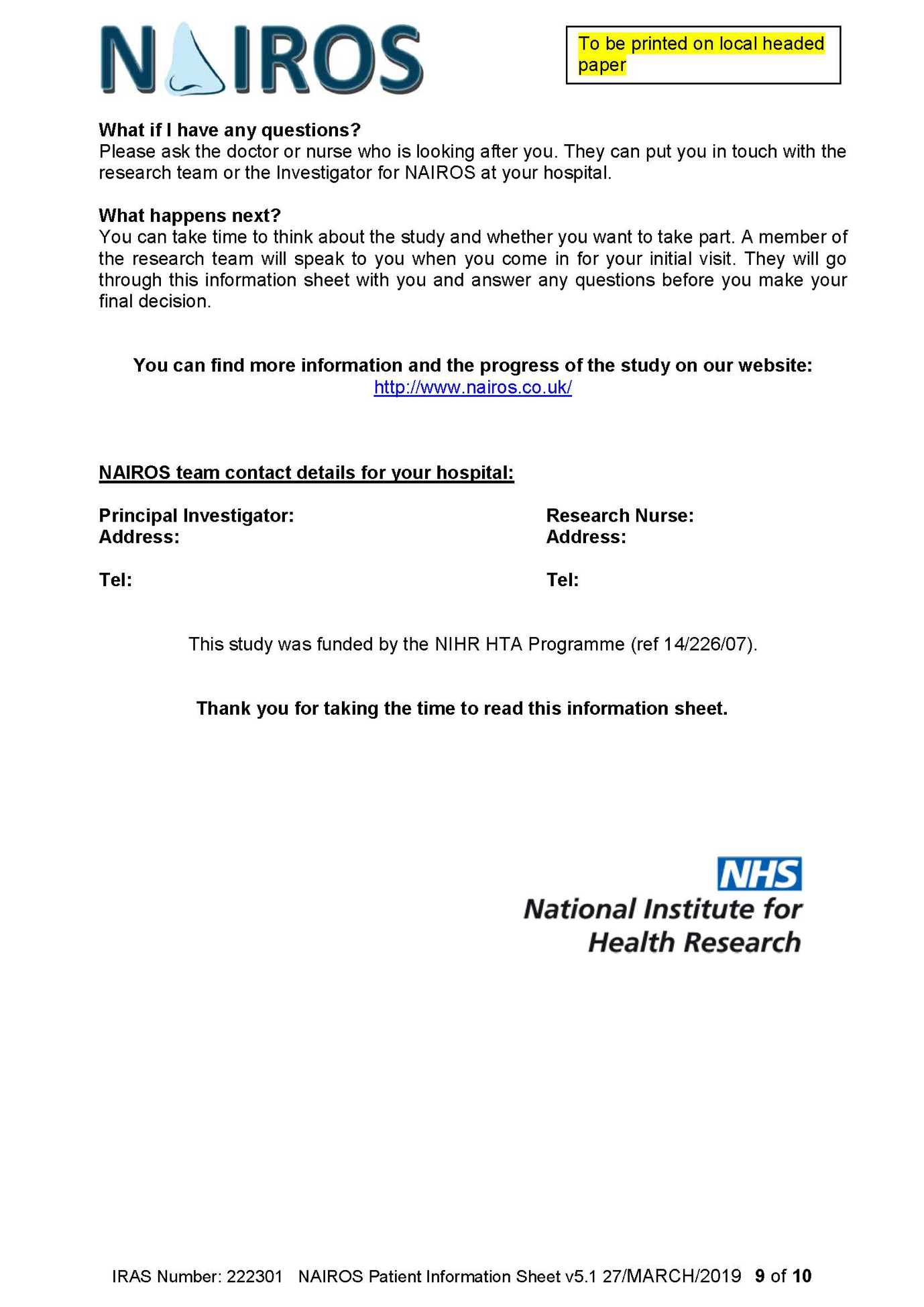
**

**
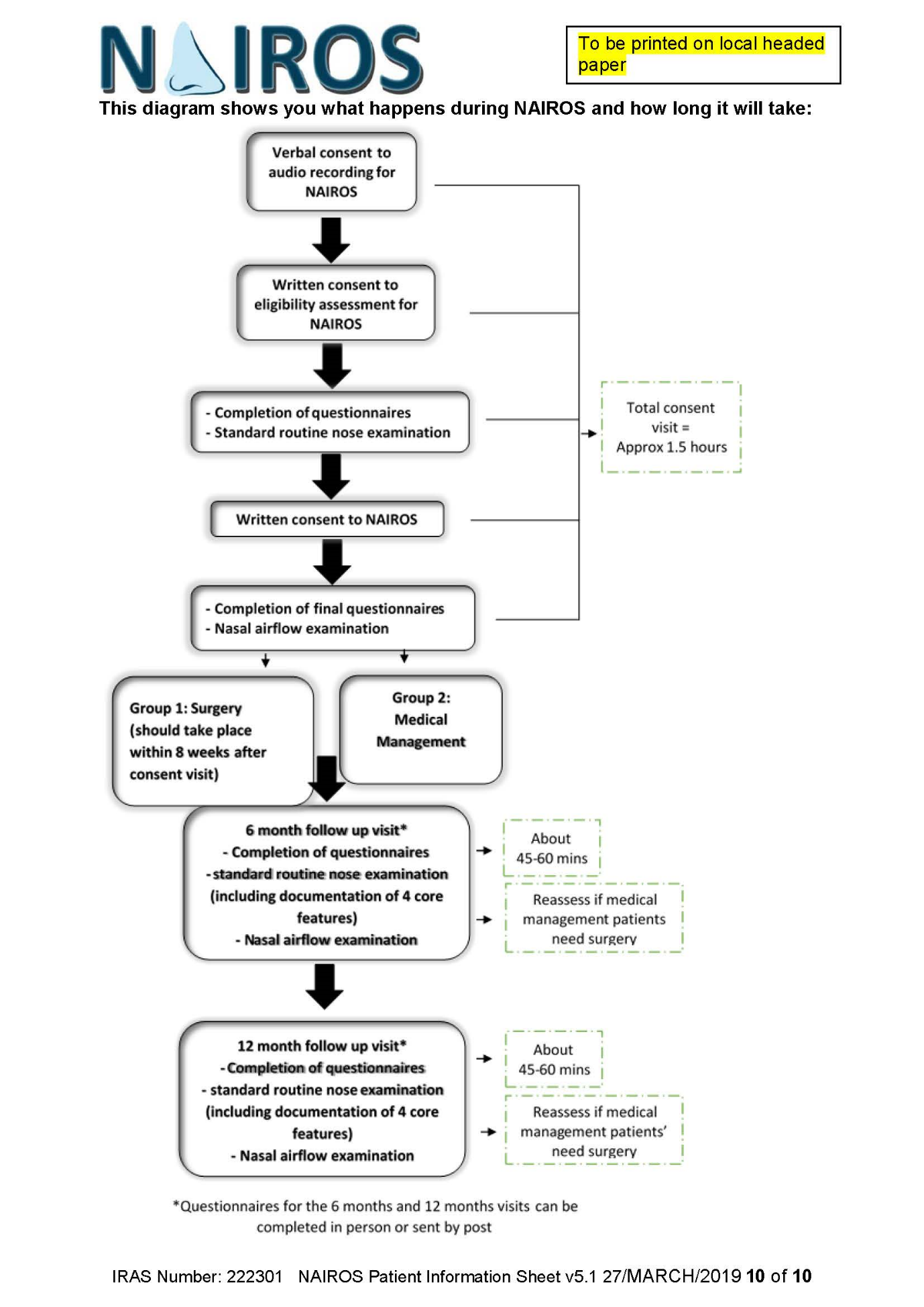
**

Supplement: Supplementary file 1 — Additional file 1. Patient Information Sheet, V5.1 dated 27 March 2019. [file 13063_2020_4081_MOESM1_ESM.docx]

**Appendix 2. NAIROS Main Informed Consent Form, V3.0 dated 16 JAN 2019**

**
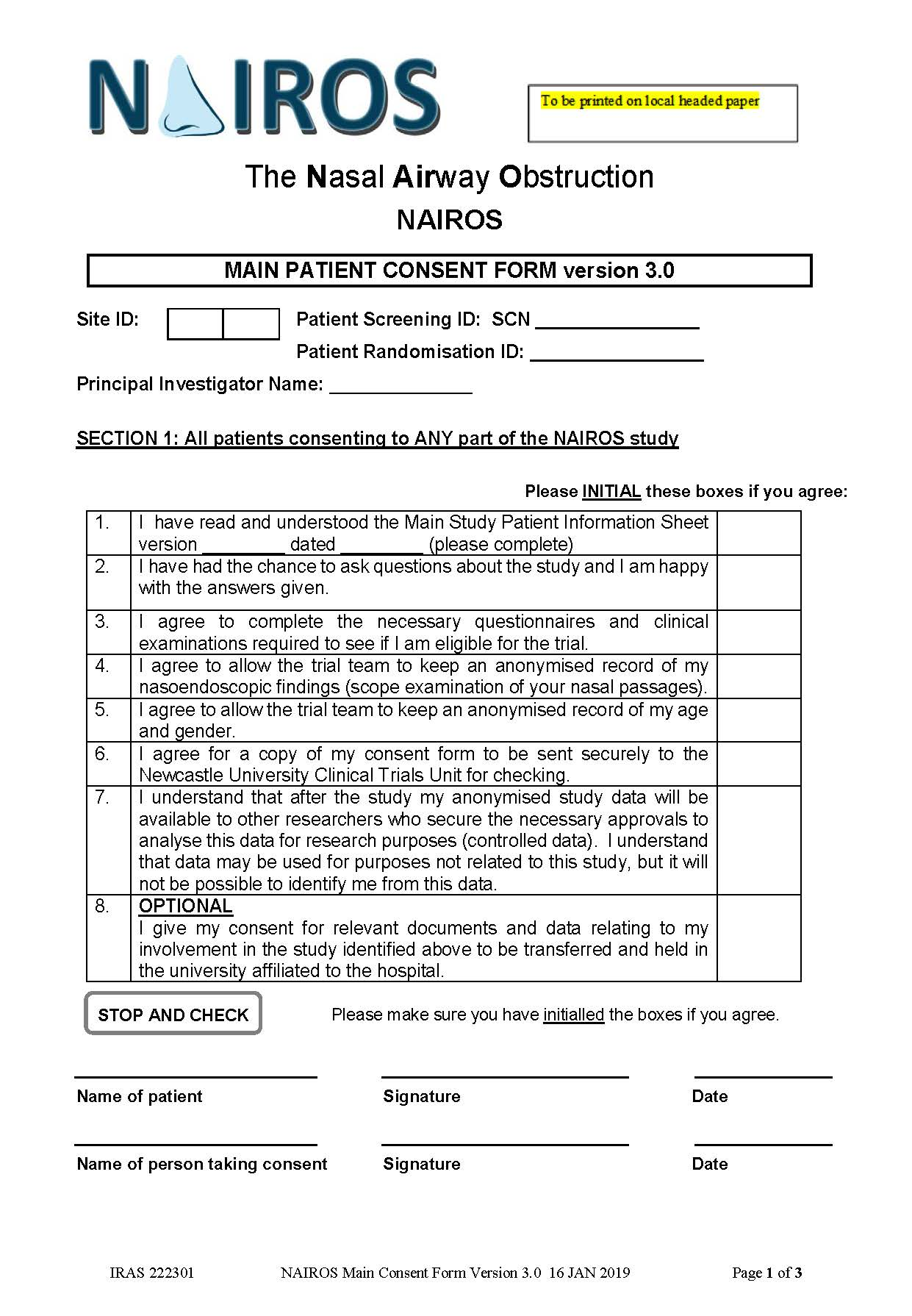
**

**
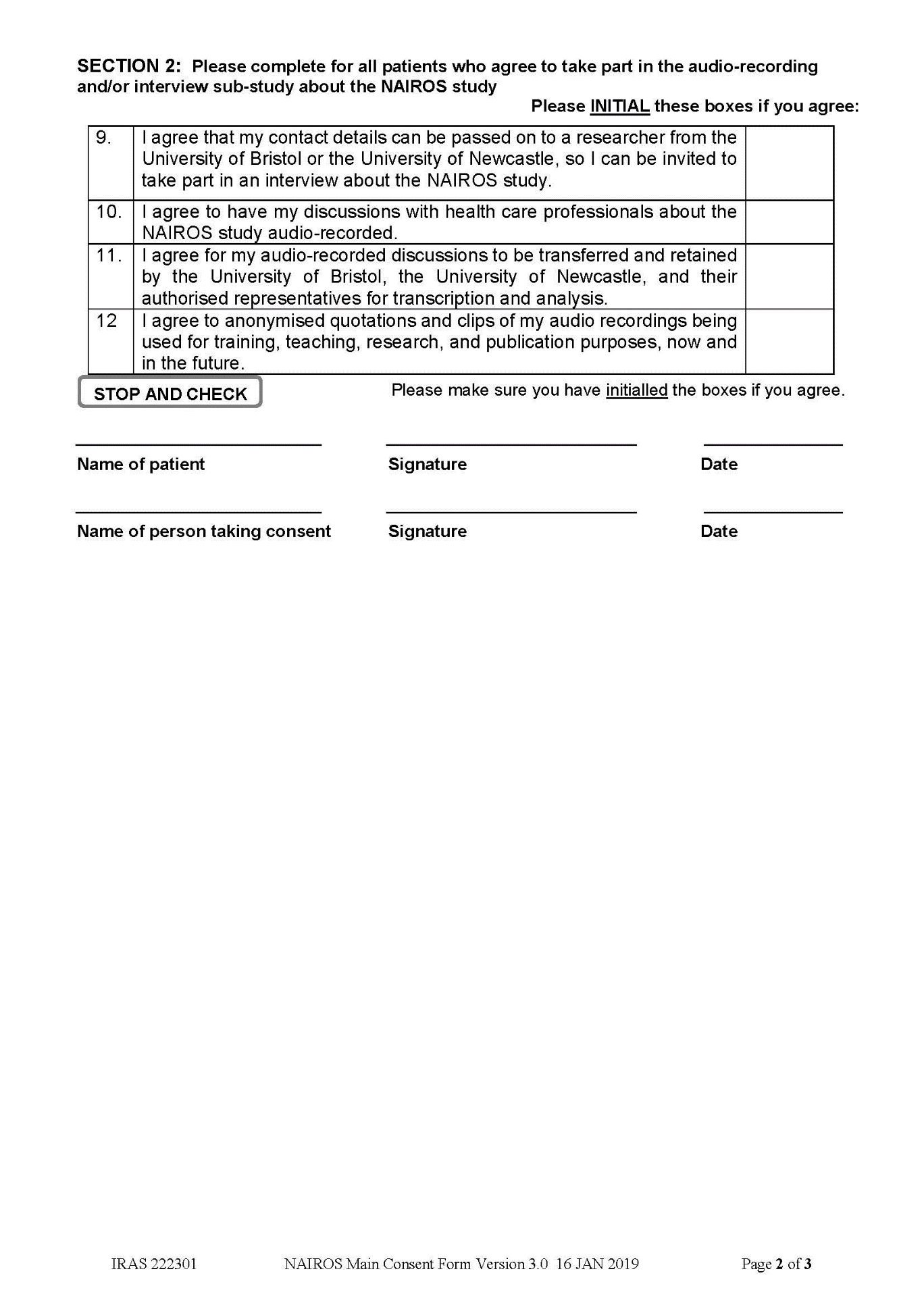
**


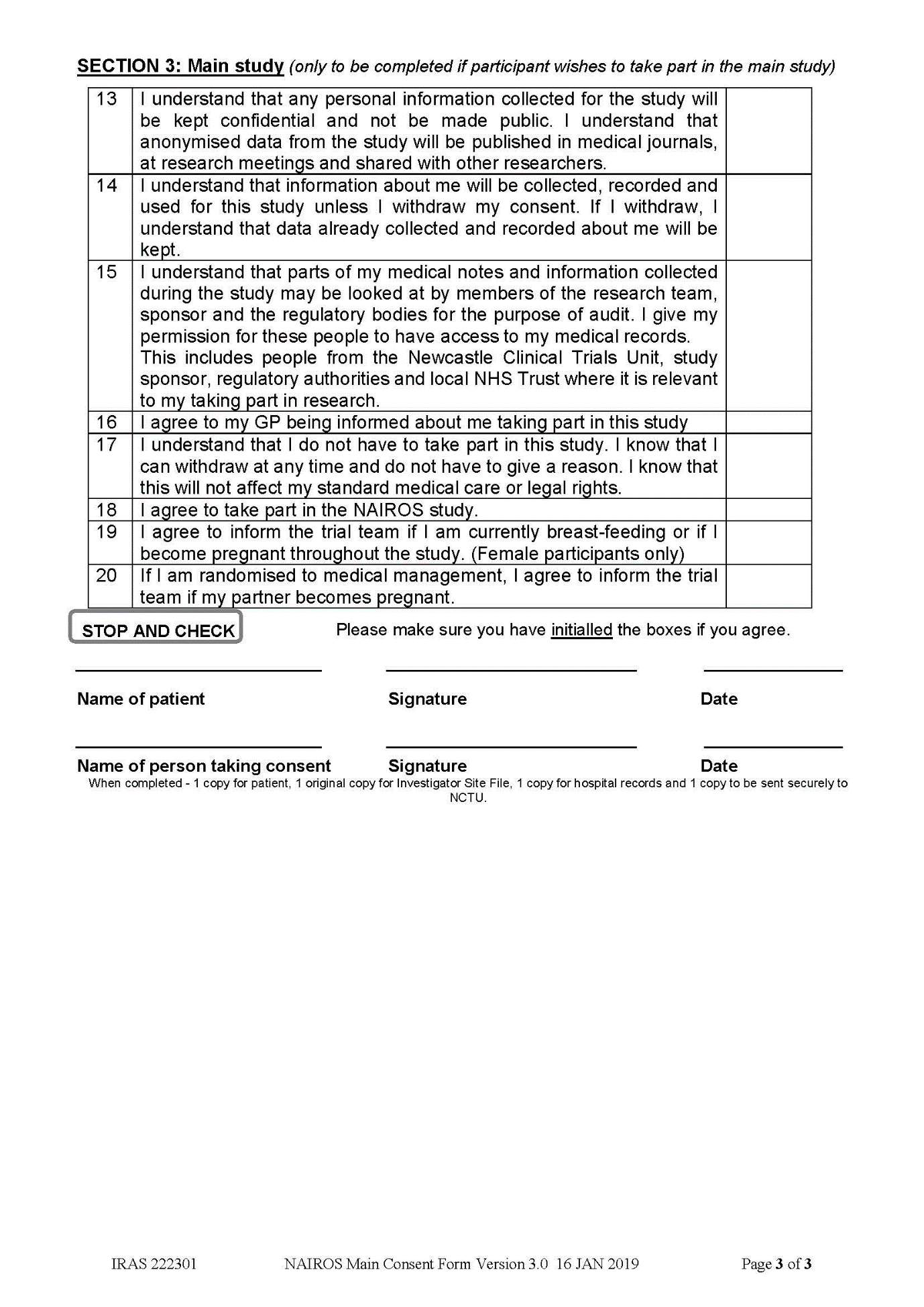

Supplement: Supplementary file 2 — Additional file 2. Nasal Airway Obstruction Study (NAIROS) Main Informed Consent Form, V3.0 dated 16 Jan 2019. [file 13063_2020_4081_MOESM2_ESM.docx]
